# PheSeq, a Bayesian deep learning model to enhance and interpret the gene-disease association studies

**DOI:** 10.1186/s13073-024-01330-7

**Published:** 2024-04-16

**Authors:** Xinzhi Yao, Sizhuo Ouyang, Yulong Lian, Qianqian Peng, Xionghui Zhou, Feier Huang, Xuehai Hu, Feng Shi, Jingbo Xia

**Affiliations:** 1 College of Informatics, Hubei Key Laboratory of Agricultural Bioinformatics, Huazhong Agricultural University, Wuhan, China; 2 Hubei Key Laboratory of Agricultural Bioinformatics, Huazhong Agricultural University, Wuhan, China; 3 College of Science, Huazhong Agricultural University, Wuhan, China; 4 College of Life Science and Technology, Huazhong Agricultural University, Wuhan, China

**Keywords:** Data fusion, Embedding data, *p*-value, Associated genes

## Abstract

Despite the abundance of genotype-phenotype association studies, the resulting association outcomes often lack robustness and interpretations. To address these challenges, we introduce PheSeq, a Bayesian deep learning model that enhances and interprets association studies through the integration and perception of phenotype descriptions. By implementing the PheSeq model in three case studies on Alzheimer’s disease, breast cancer, and lung cancer, we identify 1024 priority genes for Alzheimer’s disease and 818 and 566 genes for breast cancer and lung cancer, respectively. Benefiting from data fusion, these findings represent moderate positive rates, high recall rates, and interpretation in gene-disease association studies.

**Supplementary Information:**

The online version contains supplementary material available at 10.1186/s13073-024-01330-7.

## Background

In the scenario of genotype-phenotype association, the association significance usually comes from a good variety of sequence analysis experiments in the form of a *p*-value, e.g., GWAS [1], PheWAS [2], RNA-seq [3], and MeRIP-seq [4]. While *p*-value data provide a genome-wide significance for each genotype-phenotype association, the characteristics of high density and uncertainty make it a fine-grained but weak signal. The uncertainty of the *p*-value and the rigorous significance threshold setting [5, 6] make it challenging to obtain robust association results, and in the meantime, the interpretations of the results are largely unclear [7].

In recent years, a big trend has emerged in the field of deep learning (DL), focusing on diverse multi-omics data including genome, transcriptome, epigenome, proteome, exposome, and microbiome [8]. Commonly employed deep learning techniques have been widely utilized for feature extraction, integrated analysis, and robust predictive modeling across various life omics datasets. However, this research directs its primary attention to the perceptual aspects of deep learning methods pertaining to gene-disease association, particularly those derived from textual evidence and network structures. The primary objective of this study is to enhance the robustness and interpretability of gene-disease associations through the integration of external text and network data, thus augmenting the findings obtained from single-omic sequence analysis. In this regard, it is necessary to fuse association information from two different modalities, the association significance (*p*-values) and DL-generated phenotype descriptions (embeddings), through a data fusion strategy.

DL models have shown a strong ability to perceive semantic interpretation in texts and the topological structure of networks in association studies [9, 10]. Benefiting from its powerful perception ability, DL plays a role in interpreting the text or network data for associations and making the association prediction evidence-supportive and ontology-normalized [11, 12]. The Human Phenotype Ontology (HPO) was initially published in 2008 [13] with the goal of integrating phenotypic data for translational research and diagnostics, and it maintains a stable rate of update [14–16]. In recent years, the range of HPO has been extended from rare to common human disease [17]. Today, HPO terms are extended to a broader range of diseases [18], as well as specific diseases like cancer, as it permits the tagging and curation of the underlying phenotypes that are associated with variants described in the literature [19]. Galer et al. [20] used the terms defined in HPO, performed a DL-based semantic similarity analysis, and associated clinical features with distinct genetic etiologies. Greene et al. [21] merged HPO-coded profiles with functional gene-specific information and successfully identified several true gene-disease associations among a large collection of genome-sequenced and HPO-coded cases with rare diseases. Peterson et al. [22] derived HPO-based phenotype descriptions from patients’ clinical notes and used DL-based means to prioritize disease-associated patients. James et al. [23] systematically integrated clinical phenotype data with genotype information, and leveraged HPO-based patient phenotype and variant data into clinical variant prioritization.

In addition to data perception, the ultimate objective of this study is to comprehend the interrelationships between two distinct modalities of association information. To achieve the goal of association inference, one commonly used algorithmic option is the Bayesian network framework [24–26]. This framework is rooted in a statistical model that effectively captures and models the uncertainty of observations, enabling inference of the hidden relational dependencies within the data. With its Bayesian nature, a Bayesian network treats the data in the form of distribution and regards data relation dependencies as conditional probability [27]. Under a Bayesian framework, a Bayesian network learns the data relationship, unveils the potential conditional dependencies, and achieves relational modeling among observations, thus making it a strong tool in association investigation [28]. Shaw and Campbell [29] used the Bayesian network to combine gene variation frequency with biological annotations and developed a variation interpretation model. Dai [30] proposed IGESS, a Bayesian network framework, to model the distribution dependency of the *p*-value in GWAS and trait output, and improved the accuracy of risk variant inference. De et al. [31] proposed a Bayesian network model that aggregated inputs from multiple variant prioritization algorithms with genomic and clinical database annotations and prioritized potentially damaged genes and candidate diseases. Zhou [32] applied another Bayesian network framework to investigate the potential dependencies between GWAS summary data and mutation descriptors from the literature and reconstructed the observation and their dependencies to promote the inference of gene-disease associations. By using the Bayesian network, all of the above works facilitated conditional dependency modeling in genotype-phenotype association studies, but they ignored the perception of the data semantics as done by DL methods, thus imposing a limitation on result interpretation.

Recently, there has been a trend of hybrid strategy that combines DL with a Bayesian network, known as Bayesian deep learning (BDL), formed by a series of works [33–35]. Inheriting the Bayesian idea of traditional Bayesian neural networks (BNN), [36, 37] BDL uses the probabilistic graphical model (PGM) in the Bayesian network to model the uncertainty and relational dependence among data but integrates a DL perception module into the probabilistic graphical model through a hinge variable. A general BDL framework involves a DL perception module and a PGM inference module, which extracts high-quality semantic representations upon the observations and investigates the potential dependencies among the data. Adam [38] used a variational autoencoder (VAE) module to perceive a semantic representation from the integrated omics data including transcriptomic and proteomic data. The observation representation is then input into a Bayesian network to assist the inference of the hidden relationships among the single cell and its corresponding omics data. This work also demonstrates an intuition that the perception of omics data using DL can boost the performance of higher-level inference and in turn, the feedback from the inference process also enhances the perceptual power.

All the above advances suggest that a BDL framework not only perceives the data feature representation but also infers the hidden relationships among the data. Therefore, BDL encourages the effective synergy of DL and Bayesian network, and supports conditional dependence modeling in the genotype-phenotype association study.

This study presents a novel Bayesian deep learning model named PheSeq, which aims to bridge the phenotype descriptions with association significance in gene-disease associations. To achieve this, PheSeq trains a 96-layer deep learning module to perceive the phenotype descriptions in the literature and network, incorporates association significance within a Bayesian network framework, learns the inherent dependencies among associations through data fusion techniques, and ultimately discovers novel gene-disease observations. As a result, PheSeq offers an interpretable high-level inference for novel gene-disease associations

The PheSeq model is employed in three distinct gene-disease association case studies. The first case study involves the use of GWAS summary data for AD, the second case study utilizes transcriptomic data for breast cancer (BC), and the third one employs methylation data for lung cancer (LC). We collect *p*-values for sequence analysis under three distinct cases to obtain association significance between gene and disease. Simultaneously, we collect phenotype descriptions for each gene-disease pair in literature and network and employ a computational pipeline to generate phenotypic embedding for the gene-disease pair. Our model, utilizing data fusion learning, integrates association information from two different modalities, resulting in a more comprehensive recommendation of gene-disease associations. The findings indicate that PheSeq produces prioritized genes with a moderately positive rate when compared to traditional single sequence analysis. In the case of AD, the percentage of prioritized significant genes over background GWAS genes is 5.6%, which represents a substantial improvement over the low positive rate of 1.7% observed in the GWAS experiment. Similarly, PheSeq filters 2.3% of genes in LC methylation data and 0.75% in BC transcriptome data, compared to the respective positive rates of 4.7% and 2.7% in the conventional sequence analysis. In all three case studies, the top 50 prioritized genes include over half that is consistent with previously known gene-disease associations recorded in the DISEASES database [39]. In addition, it is worth noting that a significant proportion of prioritized genes in AD cases, specifically 90% (45 out of 50), can be readily interpreted with supporting evidence obtained from GWAS experiments or established databases.

The contribution of the PheSeq model is twofold. Firstly, it employs a data fusion strategy to improve the study of gene-disease associations by combining *p*-value data and phenotypic embeddings. Additionally, it utilizes phenotype descriptions to interpret the associations. Results in case studies show that PheSeq obtains a moderate positive rate and high recall rate, benefiting from the data fusion strategy. In addition, The model derives a vast dataset of association evidence, making it possible for the interpretation and exploration of gene-disease associations.

## Methods

### Data collection for sequence analysis, literature, and network data in three case studies

To investigate the efficacy and robustness of the PheSeq model, three diseases characterized by distinct pathological features are selected as case studies. Relevant sequence analysis and literature data are collected for each of them.

The three selected diseases, along with their corresponding sequence analysis data, represent a broad spectrum of gene-pathology associations among patient populations. Alzheimer’s disease (AD), a prototypical genetic disorder, is analyzed using genome-wide association study (GWAS) summary data to elucidate the significance of single nucleotide polymorphisms (SNPs) in genetic inheritance. Breast cancer (BC), a typical cancer type that has been extensively studied, has comprehensive and representative expression profile data for association research. We therefore employ transcriptome data for sequence analysis. Additionally, the etiology of lung cancer (LC) is more complex, influenced by environmental factors and epigenetics. Therefore, methylation data is chosen as the representative sequence analysis data.

**Fig. 1:**
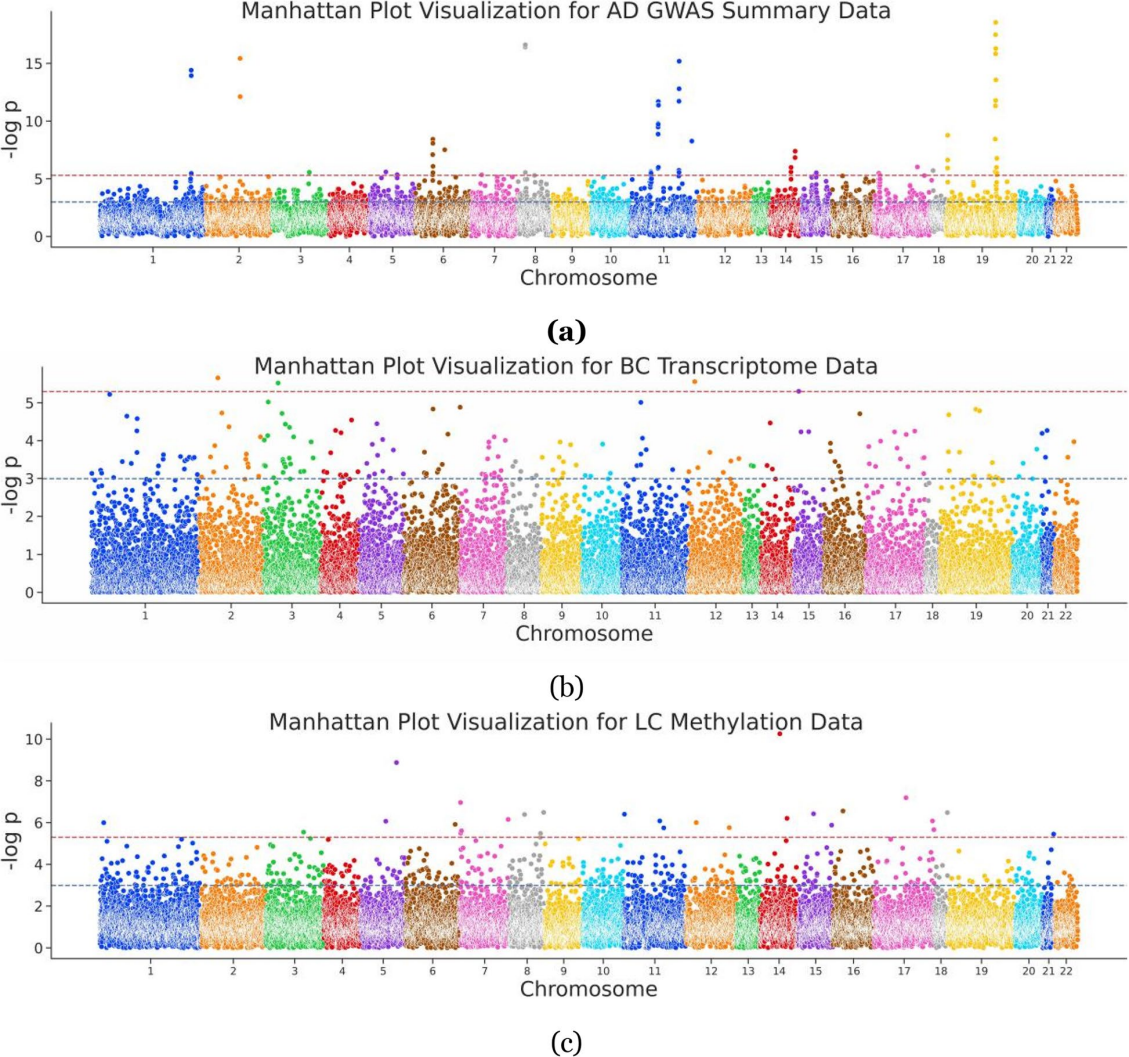
Manhattan plot of sequence analysis data. **a** AD GWAS, depicting 18,157 genes visualized in a Manhattan plot with $-\log$ *p* value represented along a vertical line and chromosome position on the *x*-axis; **b** BC sequence analysis incorporating transcriptomic data, 17,374 genes encompassed; **c** LC sequence analysis integrating methylation data, involving 24,578 genes

For the sequence analysis, the AD-related GWAS summary data were collected from GCST002245 [40] on the GWAS Catalog. The transcriptome data were generated using the Agilent G4502A_07_3 platform, and the methylation data from the Human Methylation 450 platform were retrieved from TCGA [41]. Then, the association significance for each gene was obtained, and the Manhattan-style plot of the above results is shown in Fig. 1.

In detail, for AD, GWAS summary data were downloaded from summary data of the international genomics of Alzheimer’s project (IGAP) [40] (https://www.niagads.org/datasets/ng00036), which performed a two-stage GWAS on individuals of European ancestry on 7,055,881 SNPs, and 23 genes were proposed to include the AD-related SNPs. In accordance with standard conventions [42], we adhered to a straightforward practice in retrieving genes located ± 100 kb of the SNP site by using Bedtools, thus assigning association significance to corresponding genes. For BC, the transcriptome data from TCGA [41] were collected (Platform: AgilentG4502A_07_3), which includes 597 cancer samples and 64 healthy samples. The two-sample *T*-test was used to calculate the differential expression for each gene in the patient population and the normal population, thus obtaining the association significance for each gene. For LC data, 862 samples with prognostic information were collected from TCGA (Platform: HumanMethylation450). A prognostic analysis was performed by combining the prognostic data of each patient. A Cox regression was performed to infer the correlation between the methylation site and the prognostic risk, and the association significance for each methylation site was assigned to the corresponding gene. In total, GWAS in AD covers 18,157 genes and assigns *p*-value to each of them. Meanwhile, the count of genes in sequence analysis upon BC and LC is 17,374 and 24,578, respectively (Table 1).

**Table 1:** Data resources and the statistics of covered genes from the literature and sequence analysis

	Sequence analysis		Literature extraction	
	Gene count	Data resource	Gene count	# of literature
AD	18,157	GWAS summary data (GCST002245)	14,261	24,440
BC	17,374	Transcriptome data (AgilentG4502A_07_3)	10,498	55,638
LC	24,578	Methylation data (HumanMethylation450)	20,460	81,463

To collect phenotype descriptions, disease-related literature was downloaded on a PubMed and PMC scale. Specifically, we gathered the full names of diseases (Alzheimer’s disease, breast cancer, and lung cancer) and their corresponding MeSH terms. After downloading the PMID and PMCID list, the PubTator API was then utilized to retrieve all available abstracts and full-text articles. To ensure the relevance of the literature to the diseases, keyword matching was employed as an additional filtering step. Specifically, we required that the full name or abbreviation of the disease is mentioned at least 3 or 5 times within the abstract or full text respectively. In total, 24,440 pieces of literature were obtained within the AD topic, mentioning 14,261 genes. Likewise, 55,638 and 81,463 pieces of literature were obtained for BC and LC, covering 10,498 and 20,460 genes, respectively (Table 1).

In addition, network data were collected to capture structural information of genes. Specifically, the protein-protein interaction (PPI) network data for *Homo sapiens* were gathered from the STRING database [43], and the filtering of PPIs was performed by applying a confidence threshold of 0.7, following the guidelines provided by the STRING database. As a result, a PPI network with 359,776 edges was obtained. After mapping protein IDs to gene Entrez ID, 15,131 unique genes were included in this network.

### A phenotypic embedding generation pipeline

For a given gene-disease pair, a phenotypic embedding generation pipeline is proposed to process concept and sentence embeddings from literature and process graph embedding from network data.

When processing sentences which contain a given gene, we first annotate three types of phenotype description phrases, including biological process terms, phenotypic terms, and disease terms. These terms are then normalized by gene ontology (GO), HPO, and MeSH, respectively. In detail, OGER++ [44] is used to annotate and normalize the GO terms, PhenoTagger [45] is for the HPO terms, and PubTator is used for the gene and disease mentions. Subsequently, sentences that address phenotype description of the gene-disease association are filtered by a biomedical event extraction model [32] on AGAC corpus [46]. This model detects the biomedical events in texts, including molecular physiological activity, cell physiological activity, and interactions. Altogether, concepts and sentences for each gene are encoded by BioBERT [47] and put into a deep neural network to generate concept and sentence embeddings.

When processing network that contains a specific gene, we locate the gene within the STRING network and then apply a graph embedding method. The proposed pipeline provides a diverse range of options for embedding computation, including node2vec [48], Mashup [49], BioPlex 3.0 [50, 51], HuRI, and drug-target network [50, 52], and struct2vec [53]. In the case studies, we primarily adhere to Yue’s guideline [54] and employ struct2vec to compute the graph embedding.

Finally, a dynamic meta-embedding method, proposed by Douwe [55], is used to compute an average weight of the bio-concept embedding, sentence embedding, and graph embedding for each association. The resulting integration of these distinct embedding modalities aims to enable a robust phenotypic embedding representation of each gene-disease association.

Furthermore, to facilitate user observation of the quality of their embedding data, the corresponding visualization tools are also provided in this pipeline, and the details are given in the Additional file 1.

### PheSeq, the proposed data fusion model

#### Motive of data fusion

In this section, we introduce mathematical notations to illustrate the motive and setting of the model.

For a given disease *d*, the gene-disease association data include *p*-value data $P_g$ and embedding data $L_g$ for gene *g*, each of which is collected from multi-omics sequence analysis and text/network representation learning, respectively. The left side of the figure presents association data from two perspectives: the Manhattan plot for $P_g$ from the sequence analysis and the embedded semantic space plot for $L_g$ from the embedding generation pipeline. The Manhattan plot serves as a standard graphical representation of the association significance *p*-values between genes and diseases in sequence analysis, where the chromosome position for each *g* is placed on the *x*-axis, while the significance of $P_g$ is stated as $-\log p$ along the *y*-axis. In the embedded semantic space plot, each point signifies the phenotypic embedding associated with *g* and *d*. Typically, genes with similar semantic associations cluster together. Both the Manhattan plot and the semantic space plot provide visualizations of two modalities of data pertaining to gene-disease associations.

**Fig. 2:**
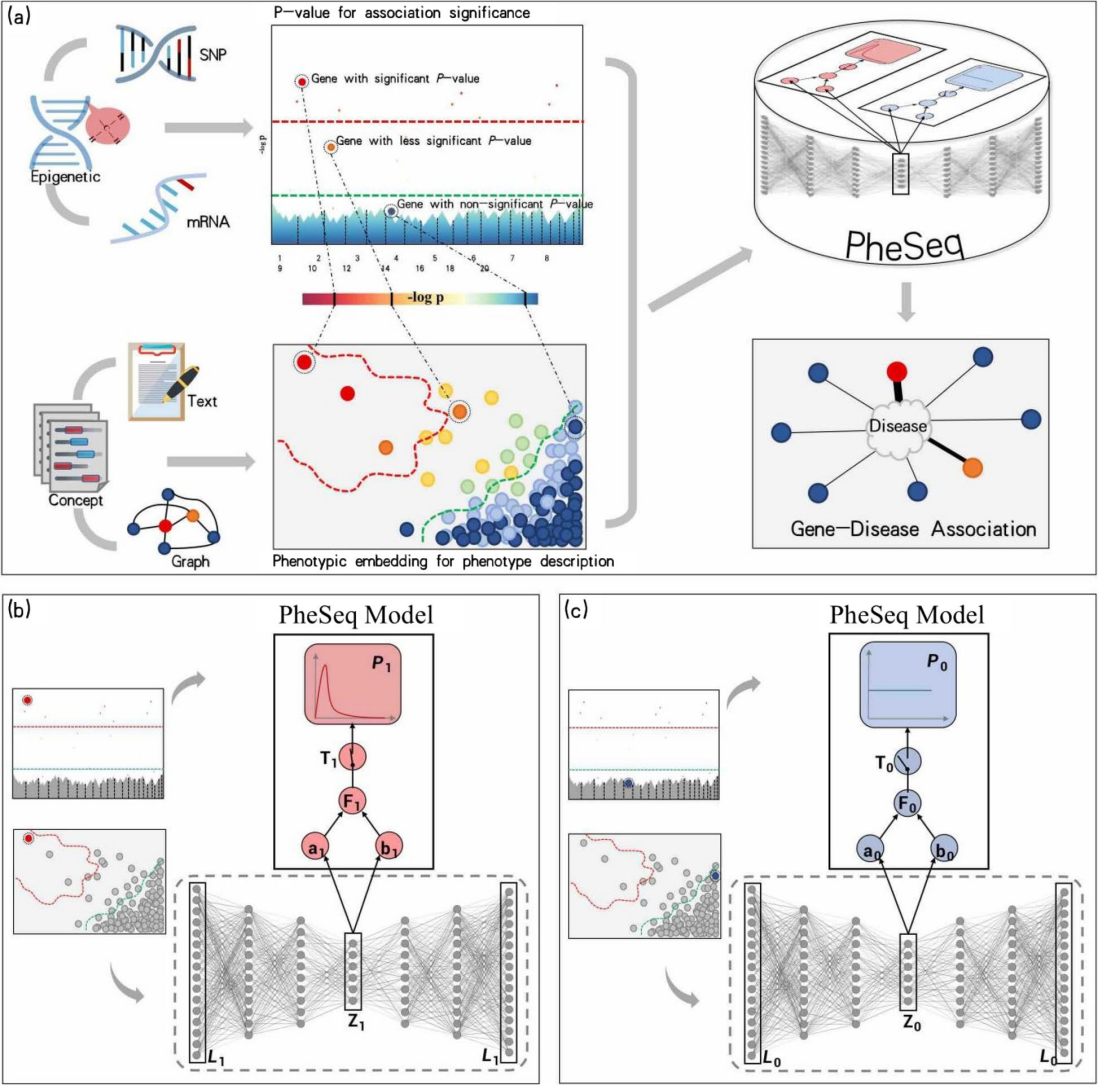
The framework of PheSeq. **a** General model input in PheSeq involves *p*-values for association significance in sequence analysis and phenotypic embeddings for phenotype description from texts or graphs. The associations with *p*-values are graphically depicted in a Manhattan-style plot. A threshold line with a strict criterion (red line) or a less strict criterion (green line) is then applied. Concurrently, a DL perception module learns the association description of gene-disease association from text or graph. Genes exhibiting significant association descriptions tend to aggregate in the top-left region of the semantic space, as shown in the figure. Analogous patterns emerge in other scenarios. Finally, PheSeq learns the data distributions and performs data fusion for gene-disease associations. **b**/**c** Data fusion of association significance and phenotype description for a significant/non-significant gene-disease association by PheSeq. For each gene-disease association, two distinct types of observations, denote as *L* for phenotypic embedding and *P* for *p*-value, are considered for data fusion. Both sets of observations are input into the PGM inference module, facilitating the learning of dependency relationships among them in conjunction with latent variables. The phenotypic embedding *L* is initially processed through the DL perception module for semantic training, resulting in the generation of high-quality embeddings denoted as *Z*. The latent variable *T* serves a pivotal role in synchronizing the phenotypic embedding data with the *p*-value data, the latter adhering to a beta distribution indicative of a predisposition toward “small-*p*-value.” In addition, another latent variable *F* functions as an association score, establishing connnections among model parameters. Conceptually, the switch mechanism activates when both the association significance and phenotype description align, effectively bridging the above heterogeneous data modalities. Part **c** shows the converse situation, wherein the data indicate non-significance for the gene-disease association. In this case, a uniform distribution is employed to characterize the distribution of the *p*-value. The remaining configurations of the model remain consistent

Generally, the *p*-value threshold or semantic similarity is applied in sequence analysis or representation learning. For example, a false discovery rate such as the Bonferroni or Benjamini test [56] is applied as a strict threshold for $P_g$-based significant analysis, while 0.05 is regarded as an empirical but less strict threshold, as shown in Fig. 2a. Therefore, the threshold of the *p*-value needs to be considered in terms of the data congruence with the phenotype description. Intuitively, from a view of semantic similarity, embedding data $L_g$ with similar significant or non-significant association semantics is prone to forming a cluster in a semantic space. As illustrated in Fig. 2a, under the guidance of a color-coding scheme, the synergy and fusion of data from these two modalities can be observed.

#### Model setting

Under a BDL framework, we propose a generative model, PheSeq, to uncover underlying gene-disease associations by bridging two types of heterogeneous association data. The data comprise the phenotype description data in texts and graphs, along with association significance data derived from sequence analysis, collectively unveiling associations between genes and diseases.

PheSeq consists of two modules, i.e., a DL module for the perception task and a PGM module for the inference task. The DL module consists of a 96-layer deep neural network, designed to perceive semantic interpretation in texts and the network topology structures related to the phenotype description of associations. In the meantime, the PGM module models the association significance with inherent uncertainty via a random variable setting and captures the distribution dependency among phenotype description and association significance. Finally, data fusion of the heterogeneous association data is performed in a BDL framework, whose inherent attribute is a generative model. This framework then generates novel association significance by establishing a connection between the two types of heterogeneous association data. In summary, PheSeq learns the data congruence of phenotype description and association significance, leverages the collective power of the heterogeneous data, and enables the inference of novel gene-disease associations.

PheSeq encompasses two algorithmic variations based on the distinction in pheotypic embedding input. Specifically, it includes a Static-PheSeq model designed for a predefined set of embedding data and a Dynamic-PheSeq model tailored for a set of flexible and learnable embedding data. The Static-PheSeq model assumes that the embedding data are already well-learned to represent the source data, so a fixed deep-learned representation is fed into a Bayesian network and captures the potential relations and dependencies among the genotype-phenotype data. Meanwhile, the Dynamic-PheSeq model involves both phenotypic embedding and *p*-value into a BDL framework, and the embedding data and all the PGM parameters are adjusted dynamically.

#### Embedding computation and *p*-value modeling for gene-disease association

In the DL perception module, a neural network $V_{\theta }(\cdot )$ is introduced to perceive the association description and learn a semantic representation. In the Static-PheSeq model, $V_{\theta }(\cdot )$ is implemented by the BioBERT deep network to generate a fixed representation. Instead, $V_{\theta }(\cdot )$ is implemented by a VAE neural network in the Dynamic-PheSeq.

To model the gene-disease association, two latent variables are imported into the PGM inference module. First, a latent variable $F=\{F_g\}$ represents the score of the gene-disease association and follows the beta distribution with $a_g$ and $b_g$ as parameters, i.e., $F_g\sim Beta(a_g, b_g)$. Another latent variable $T=\{T_g\}\sim Bernoulli(F_g)$ plays a “switch” role in synchronizing the heterogeneous association data. It takes a binary value, where 1 indicates a *g*-*d* association (as shown in Fig. 2b) and 0 indicates a non-association (as shown in Fig. 2c).

Modeling the uncertainty and prior of the *p*-value has long been a research issue [57, 58]. Parker and Rothenberg [59] found that any distribution on the interval [0, 1] can be modeled as a mixture of individual beta distributions. Allison [60] chose a standard two-parameter beta distribution for the *p*-value as it allows for the flexible modeling of shapes on the unit interval and demonstrated its effectiveness in *p*-value from microarray data. Xiang [61] further justified the precision of the parameter estimated obtained by fitting a mixture beta-uniform distribution to a *p*-value distribution. Hu [62] presented empirical evidence that a standard beta distribution can accurately capture the shape of the true density of the *p*-value. Zhou [32] adopted a beta-uniform approximation approach within the Bayesian network framework to approximate the actual *p*-value obtained from GWAS.

Therefore, in the PheSeq model, a mixture beta-uniform distribution is selected as the prior of *p*-value and we assume

$$P_g \sim T_g Beta(\alpha _g,1)+(1-T_g)U(0,1).$$

When the switch is on ($T_g=1$), $P_g$ follows a beta distribution, $Beta(\alpha _g, 1)$. Here, $\alpha _g$ is generated by $\Phi$ and prone to be close to zero, thus leading to a significant association between gene *g* and disease. Conversely, when the switch is off ($T_g=0$), $P_g$ follows a uniform distribution of *U*(0, 1) and makes it a high chance to sample a less significant *p*-value.

In summary, all the variables used in PheSeq are:

$$\begin{aligned} \left\{ \begin{array}{l} \text {Obs: } Z=\{Z_g\}, P=\{ P_g\}, g=1,\cdots ,G,\\ \text {Latent variables: } F=\{F_g\}, T=\{ T_g\}, g=1,\cdots ,G,\\ \text {Model parameters: } \Theta = \left\{ \phi ,\alpha _g,a_g,b_g \right\} , g=1,\cdots ,G. \end{array} \right. \end{aligned}$$

#### The BDL framework for Static-PheSeq model solving

In the view of the generative model, the optimization goal of Static-PheSeq is to maximize the log-likelihood of the *p*-value and random variables conditioned on phenotype descriptions. Since the DL module is fixed in Static-PheSeq when tuning the PGM module, it is straightforward to obtain the parameter iterations via the standard maximum likelihood estimate (MLE) computation.

Based on the distribution dependencies provided in Fig. 2b and c, the logarithm of the joint probability equals to

1 $$\begin{aligned} \begin{array}{l} \log p (P,T,F|Z,\Theta ) = \log \prod \limits _{g=1}^G p(P_g, T_g, F_g|Z_g, \Theta ) \\ = \sum \limits _{g=1}^G [ T_g \log \alpha _g+T_g (\alpha _g - 1 ) \log P_g +\log \frac{\Gamma (a_g+b_g)}{\Gamma (a_g)\Gamma (b_g)} ] \\ \quad + (T_g + a_g -1 )\log F_g+ (b_g-T_g)\log (1-F_g) \\ \end{array} \end{aligned}$$

Considering the variational sampling of the latent variable, the expectation of the logarithm of the joint pdf, $L(\Phi ) = E_{q(T, F)}[\log p(P, T, F | Z, \Theta )]$, is set as the loss function. A Monte Carlo estimation leads to $L_{MC}(\Phi ) = \log p(P, T^*, F^* | Z, \Theta )$, where $T^*, F^* \sim q^*(T, F)$. As $\nabla _\Phi L(\Phi ) \approx \nabla _\Phi L_{MC}(\Phi )$, the noisy estimate of the gradient with respect to the neural network parameters, $\Phi$, is

2 $$\begin{aligned} \begin{array}{l} \nabla _{\phi }L_{MC}(\phi ) = \sum \limits _{g=1}^{G} ( \nabla _{\alpha _g} \frac{\partial \alpha _g}{\partial \phi } + \nabla _{a_g} \frac{\partial a_g}{\partial \phi } + \nabla _{b_g} \frac{\partial b_g}{\partial \phi }), \\ \text {where}\\ \left\{ \begin{array}{l} \nabla _{\alpha _g}=T_g^{*}(\log P_g + \frac{1}{\alpha _g}),\\ \nabla _{a_g}=\frac{\Gamma (a_g+b_g)\Psi (a_g+b_g)-\Gamma (a_g+b_g)\Psi (a_g)}{\Gamma (a_g+b_g)}+ \log F_g^* , \\ \nabla _{b_g}=\frac{\Gamma (a_g+b_g)\Psi (a_g+b_g)-\Gamma (a_g+b_g)\Psi (b_g)}{\Gamma (a_g+b_g)} + \log (1-F_g^*). \end{array} \right. \end{array} \end{aligned}$$

The gradient computation leads to the optimization iteration in MLE. Here, $\Psi (x)=\Gamma ^\prime (x)/ \Gamma (x)$ is the digamma function. Eventually, a gradient ascent iteration, $\Phi ^{(t+1)}=\Phi ^{(t)} + \eta \nabla _\Phi L(\Phi )$, is adopted, where $\eta$ is the learning rate.

In Static-PheSeq, the model parameters are mainly derived by formula (2). The iteration ends when these parameters achieve convergence. The implementation of Static-PheSeq is concluded in Algorithm 1, “MLE for Static-PheSeq,” and the complete proof is given in Additional file 2. The effectiveness of the model is then evaluated in the selected case studies.

#### The BDL framework for Dynamic-PheSeq model solving

Compared with Static-PheSeq, the main difference in Dynamic-PheSeq is the importing of the learnable embedding data $Z_g$, which is encoded by the description data $L_g$ in a variational autoencoder (VAE) framework. It should be noted that the model parameters, *a* and *b*, are relevant to the input learned embedding *Z* and the neural network with parameter $\Phi$, thus denoting it as $a(\Phi , Z)$ and $b(\Phi , Z)$, respectively.

Since $Z_g$ and $P_g$ need to be learned jointly in Dynamic-Pheseq, a maximum a posteriori (MAP) estimation and MLE for Bayesian network optimization are applied in the model solving. Here, the optimization goal is to maximize the evidence lower bound (ELBO), which is obtained by computing the expectation of the logarithm of evidence, w.r.t. posterior of all latent variables, i.e.,

$$\begin{aligned} L(q)&= E_q[\log p(P|T)+\log p(T|F)+\log p(F|Z)\\&\quad +\log p_\theta (L|Z)] -\mathbb{K}\mathbb{L}(q_\theta (Z|L)\Vert P(Z)) \\&\quad -E_q[\log q(F)] -E_q[\log q(T)], \end{aligned}$$

where, $q_\theta (Z_g |L_g)$ denotes an approximation for the posterior pdf of embedding $Z_g$ generated from the DL perception module, and $\mathbb{K}\mathbb{L}(\cdot \Vert \cdot )$ refers to the Kullback-Leibler divergence between two pdfs.

Subsequently, MAP with respect to $q_\theta (Z_g |L_g)$ is applied to maximize the objective for $\{F_g\}$, where $\theta =\{w_{\{1...L\} }, b_{\{1...L\} } \}$ is the parameters of the DL neural network, and $w_l$ and $b_l$ are the weight and bias of the $l^{th}$ layer, respectively.

$$\begin{aligned} \begin{array}{l} L^{MAP}(F_g, T_g, \Theta , \theta )\\ =T_g(\log \alpha _g + (\alpha _g-1)\log P_g)+T_g\log F_g + (1-T_g)\\ \quad \cdot \log (1-F_g) +\log \frac{\Gamma (a_i + b_i)}{\Gamma (a_i)\Gamma (b_i)} + (a_g-1)\log F_g + (b_g\\ \quad -1)\log (1-F_g) +E_{q_\theta (Z_g|L_g)}[\log p(L_g|Z_g)] -\mathbb{K}\mathbb{L}\\ \quad (q_\theta (Z_g|L_g) \Vert p(Z_g))) - \frac{\lambda _w}{2} \sum _{l=1}^{L}(\Vert W_l \Vert _F^2+ \Vert b_l \vert _2^2). \end{array} \end{aligned}$$

By using the block coordinate ascent, direct computation yields the iteration formula for $F_g$, i.e., $F_g \leftarrow (T_g+a_g-1)/(a_g+ b_g -1)$. Considering only the terms related to $Z_g$, the Monte Carlo estimation is used to simplify the expectation calculation.

$$\begin{aligned} \tilde{L}(\Theta ,\theta ;Z_g)&= \frac{1}{N} \sum \limits _{n=1}^{N}[\log p_\theta (L_g|Z_g^{(n)})+\log p(F_g|Z_g^{(n)})] \\&\quad -\mathbb{K}\mathbb{L}(q_\theta (Z_g|L_g)\Vert P(Z_g)), \end{aligned}$$

where $Z_g^{(n)}=\mu _g+ \sigma _g+ \varepsilon _g^{(n)}$, and $\varepsilon _g ^{(n)} \sim N(0, I_K)$, and $\{1, 2, \cdots , N\}$ is the index for Monte Carlo sampling.

Given a fixed $F_g$ in the PGM, the gradient computation with respect to $\mu _g$ and $\sigma _g$ suffices to optimize the DL perception module.

3 $$\begin{aligned} \left\{ \begin{array}{l} \nabla _{\mu _g}\tilde{L}(\Theta ,\theta ;Z_g) = \frac{1}{N} \sum \limits _{n=1}^{N}[\nabla _{\mu _g} \log p_\theta (L_g|Z_g^{(n)})\\ \qquad \qquad \qquad \qquad +\nabla _{\mu _g} \log p(F_g|Z_g^{(n)})] -\mu _g,\\ \nabla _{\sigma _g}\tilde{L}(\Theta ,\theta ;Z_g) = \frac{1}{N} \sum \limits _{n=1}^{N}[\nabla _{\sigma _g} \log p_\theta (L_g|Z_g^{(n)})\\ \qquad \qquad \qquad \qquad +\nabla _{\sigma _g} \log p(F_g|Z_g^{(n)})] +\frac{K(1-\sigma _g)}{2\sigma _g}. \end{array} \right. \end{aligned}$$

From (3), optimization of the weights and bias in DL perception modules is performed through a conventional backpropagation process. The gradient ascent iteration, $\mu _g^{(t+1)} = \mu _g^{(t)} + \eta \nabla _{\mu _g} L(\Theta , \theta ; Z_g)$ and $\sigma _g^{(t+1)} = \sigma _g^{(t)} + \eta \nabla _{\sigma _g} L(\Theta , \theta ; Z_g)$, is applied, where $\eta$ is the learning rate.

A prerequisite of MLE computation is to compute the joint probability of all observations and latent variables conditioning on parameters set, $\Theta$. From the graphical model in Fig. 2b and c, we have the logarithm of the joint probability

4 $$\begin{aligned} \log p{} & {} (P,T,F,Z,L |\Theta )\nonumber \\{} & {} =\log \prod\nolimits _{g=1}^{G} p(P_g, T_g, F_g, Z_g, L_g|\Theta )\nonumber \\{} & {} =\sum\nolimits _{g=1}^{G} [ T_g \log \alpha _g+T_g \left( \alpha _g - 1 \right) \log P_g\nonumber \\{} & {} \quad + \left( T_g + a_g -1 \right) \log F_g + \left( b_g-T_g \right) \log \left( 1-F_g\right) \nonumber \\{} & {} \quad +\log \frac{\Gamma \left( a_g+b_g\right) }{\Gamma \left( a_g\right) \Gamma \left( b_g\right) }+\log p_\theta (L_g|Z_g) + \log p(Z_g)]. \end{aligned}$$

Similar to Static-PheSeq, the expectation of the logarithm of the joint probability w.r.t. posterior distribution of the latent variables is set up as the loss function, i.e., $L(\Phi ) = E_{q(T, F)}[\log p(P, T, F, Z, L|\Theta )]$

The gradient computation w.r.t. $\Phi$ considers the back-propagation flows through the model parameters *a*, *b*, and $\alpha$, and the iteration formula is the same as the Static-PheSeq in (2). The implementation of Dynamic-PheSeq is concluded in Algorithm 2, “MAP-MLE for Dynamic-PheSeq,” and the complete proof is given in Additional file 2.

## Results

The results section is organized based on the following logic. Firstly, we present the obtained association significance data and phenotype description data for three different case studies, visualizing the distribution consistency required for data fusion. Secondly, we evaluate the data fusion results of the PheSeq model, including its performance on the reference dataset DISEASES [39] and a quantitative comparison analysis with results obtained from a single sequence analysis method. Subsequently, we provide an overall comparative observation of the predictions of PheSeq and a single sequence analysis method. Following that, we analyze the positive impact imposed by phenotype description in the PheSeq model. Subsequently, considering that PheSeq incorporates prior knowledge from the literature, we design ablation study to assess PheSeq’s predictive capability by removing prior knowledge. Simultaneously, we conduct a horizontal comparison between PheSeq and several other data fusion methods, comparing the differences in data modalities and data integration strategies. Finally, we develop a phenotype description network to exemplify and showcase the results.

### Data visualization for association significance and phenotype description

**Fig. 3:**
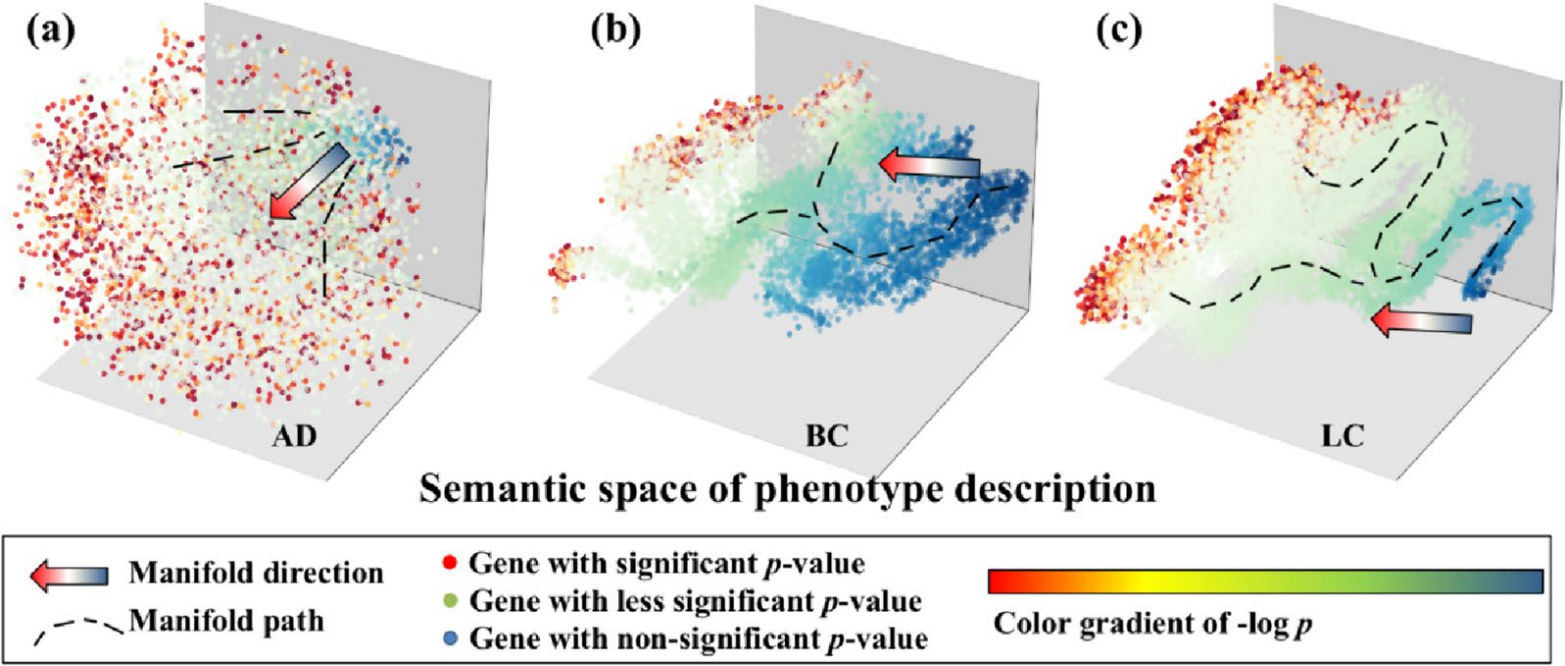
View of data congruence in three case studies. **a** 3-D semantic representation of AD genes; **b** BC genes with 3-D representation; **c** LC genes with 3-D representation. With the color gradient representing the significance level by a single sequence analysis, genes after the phenotypic embedding computation are projected onto a 3-D semantic space. Intuitively, the significant and less significant disease-associated genes are distinguished along the manifold direction based on their phenotypic embeddings. The observation suggests the high data quality of association significance and phenotype description, which supports the subsequent data fusion

In the context of three distinct case studies, a total of 24,440 AD-related literature, 55,638 BC-related literature, and 81,463 LC-related literature are fed into the phenotypic embedding generation pipeline. This yields 18,157 gene-AD pairs, 17,374 gene-BC pairs, and 24,578 gene-LC pairs, respectively. We visually represent these associations within the cubic grid in the graphical presentation in Fig. 3. Leveraging the inherent principles of semantic computation, gene-disease pairs with similar phenotypic descriptions are anticipated to exhibit proximity within this embedding space.

To observe the data congruence of the phenotypic embedding and *p*-value from sequence analysis, we employ a color-coding scheme to visualize the congruence in the distribution of two distinct modalities of data. Here, each gene is colored in a gradient ranging from red to blue, with color intensity denotes the level of statistical significance associated with the *p*-value of the corresponding gene.

In this figure, the congruence in data distribution between association significance and phenotype description is readily discernible through distinct data partitioning and segmentation. Specifically, genes exhibiting significant *p*-values (depicted as red dots) tend to disperse across the outer regions of the 3-D manifold space along the manifold path. Conversely, genes with non-significant *p*-values (represented by green-blue dots) manifest discernible partitioning and segmentation along a distinct direction within the manifold space.

In summary, the significance of *p*-values aligns with the clustering trend observed in phenotypic embedding. This suggests the potential and rationale for merging embedding data with significant *p*-values to prioritize disease-related genes. Furthermore, this fusion-based approach has the potential to deepen our understanding of gene-disease associations with the aid of phenotype descriptions.

### Evaluation of the predicted genes by PheSeq

After feeding the association significance data and phenotype description data into the PheSeq model in AD, BC, and LC cases, model iterations ran data fusion processes and generated new *p*-values for gene-disease associations. Upon the generation of the association significance for each gene after PheSeq implementation, abundant novel gene-disease associations were subsequently suggested.

#### Comparison of prioritized genes by PheSeq and sequence analysis methods

As illustrated in Table 2, the number of genes predicted by the PheSeq model for AD is 1024. This accounts for 5.6% of the 18,157 background GWAS genes, thereby establishing a moderate ratio when compared to the low positive rate of 1.7% obtained from the GWAS experiment. Similarly, the PheSeq model prioritizes 818 BC genes with a positive rate of 4.7%, which is comparatively higher than the positive rate of 2.7% obtained from the transcriptome experiment utilizing AgilentG4502A_07_3. Furthermore, 566 genes are prioritized for LC, and the resulting positive rate of 2.3% is significantly higher than the positive rate of 0.75% in the methylation experiment with Human Methylation 450.

**Table 2:** Gene-disease association discovery in the case studies via PheSeq and sequence analysis

	Significant genes by PheSeq		Significant genes by sequence analysis		# Genes overlapped
	# Gene	Ratio	# Gene	Ratio	
AD	1,024	$\frac{1,024}{18,157}=5.6\%$	311	$\frac{311}{18,157}=1.7\%$	236
BC	818	$\frac{818}{17,374}=4.7\%$	470	$\frac{470}{17,374}=2.7\%$	347
LC	566	$\frac{566}{24,578}=2.3\%$	184	$\frac{184}{24,578}=0.75\%$	68

PheSeq yields newly predicted genes for the association study, of which, a good portion are overlapped ones with single sequence analysis, and the rest are newly recalled ones. In summary, for AD, 236 genes out of 1024 prioritized genes overlap with the GWAS experiment. Similarly, 68 out of 566 genes in LC and 347 out of 818 genes in BC overlap with the methylation experiment and transcriptome experiment, respectively. Furthermore, PheSeq recalls 768, 471, and 498 novel significant genes for AD, BC, and LC, respectively.

#### Evaluation by the benchmark dataset

To evaluate the prioritization result, a benchmark database, DISEASES [39], is referenced, which integrates 36,448 gene-disease associations from three resources with increasing reliability, i.e., “Text mining,” “Experiments,” and “Knowledge.” Among them, the “Text mining” results are retrieved by text co-occurrence, and “Experiments” collect GWAS databases like target illumination GWAS analytics (TIGA), Catalogue of Somatic Mutations in Cancer (COSMIC), and DistiLD, while the “Knowledge” results involve general gene-disease association databases like AmyCo, MedlinePlus, and UniProtKB.

In AD, three mutually overlapping sources in DISEASES, Text mining, Experiment, and Knowledge, encompass 315, 339, and 26 significant genes, respectively, corresponding to 624 AD genes in total. Among these, PheSeq achieves 128, 48, and 17 gene hits in the three sources, respectively, contributing to 151 significant AD genes in total. The result reveals a relatively higher recall rate, i.e., 17/26, in the Knowledge source, lower in the Experiment source (48/339), and intermediate in the Text mining source (128/315).

The recall rate in BC and LC is higher than that in AD. In BC, the three sources contain 176, 344, and 29 LC genes, contributing to 533 LC genes. The recall rate for each source is 72/176, 73/344, and 19/29. The overall recall rate is 159/533, whereas in LC, the overall recall rate is 342/669. PheSeq hit 119 out of 297 genes in the Text mining source, 239 out of 391 genes in the Experiment source, and 21 out of 25 genes in the Knowledge source. In brief, though it still misses many hits in DISEASES, PheSeq obtains a reasonable recall rate.

**Fig. 4:**
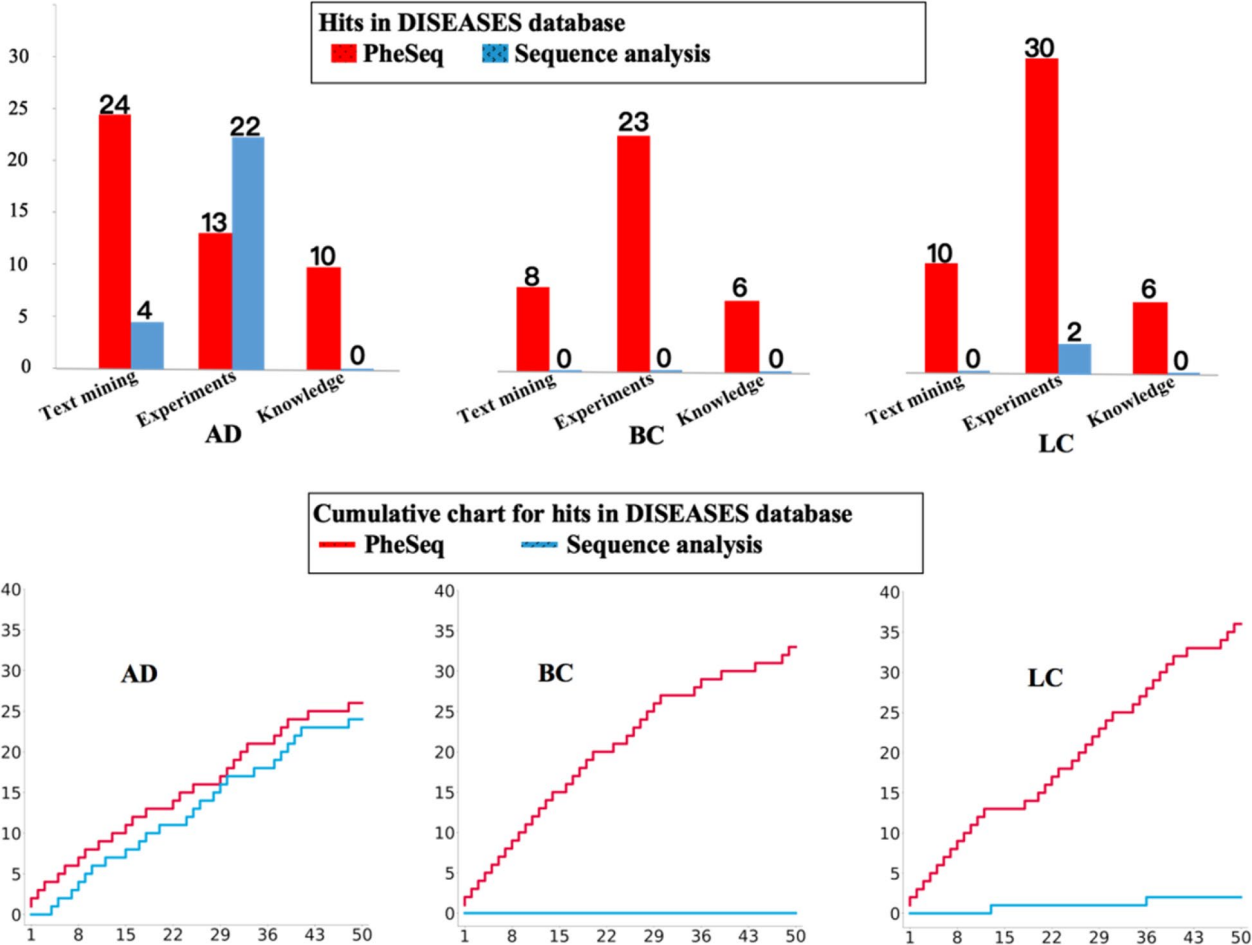
The top prioritized genes from PheSeq and sequence analysis in the DISEASES database. The hits plot and cumulative charts in DISEASES serve to compare the recall rate of PheSeq and sequence analysis methods

By observing the Top 50 genes prioritized by PheSeq (Fig. 4), it is concluded that over half of them have been recorded in the DISEASES database, of which 26/50 for AD, 33/50 for BC, and 36/50 for LC. The high coverage reveals that PheSeq well replicates the associations in the benchmark database. In comparison, the record of significant genes by sequence analysis is relatively scarce within the top 50. It is noted that DISEASES covers a good portion of the significant genes by sequence analysis. Among 311 significant AD genes through sequence analysis, 233 are encompassed in the DISEASES database. In the cases of sequence analysis experiments for BC and LC, 424 out of 470 significant BC genes and 137 out of 184 significant LC genes are cataloged in the DISEASES database.

Interestingly, PheSeq replicates the associations in the three DISEASES resources with different coverage rates. Among them, 24 genes in the AD prediction have been recorded in the “Text mining” part, while the number for BC and LC is 20 and 10. Comparatively, LC obtains higher recall in the “Experiments” part. The results also suggest that it is hard for a single source of data to recover gene-disease associations, and PheSeq is capable of fusing the heterogeneous data to achieve better data comprehension.

#### Investigation of top prioritized AD-associated genes

Taking AD as an example, the top 5 genes with and without GWAS supports are investigated. Being the Top 5, MAPT, PSEN1, APP, APOE, and GRN are known vital ones in the AD pathological hypothesis. In detail, MAPT encodes the Tau protein, and its hyper-phosphorylation forms the neurogenic fiber tangles in neurons and leads to neuronal apoptosis [63]. Moreover, mutations in PSEN1 and PSEN2 have an impact on an APP-cleaving enzyme, $\gamma$-secretase, thus regulating APP expression. Meanwhile, the accumulation of Abeta, an APP-encoded protein, forms the fibrillar amyloid plaques in the brain and impairs the ability of spatial learning and memory, which is a known direct cause of AD [64]. Being the most widely studied AD-associated gene, APOE is known to cause neuro-inflammation among AD patients by affecting the microglia [65]. In addition, GRN is a causal gene for frontotemporal dementia, a neurodegenerative disease [66].

Investigation of the top 50 genes leads to a discovery that a good trade-off leverages association significance and phenotype description and helps to infer the potential associations. First, 24 out of 50 prioritized PheSeq genes pass the IGAP GWAS significant test. They both carry significant *p*-values and supportive semantic evidence. For example, TREM106B, with a significant *p*-value of 9.53E−14, wins 50 hits in the literature. In addition, 18 out of 50 prioritized genes pass the significant test in the GWAS dataset EFO_0000249. Among them are GRN, TMEM106B, SPI1, CR1, and PICALM from GCST90044699; SORL1 and SQSTM1 from GCST002245; CLU and ABCA7 from GCST90012877; MAPT from GCST90038452; APP from GCST012182; APOE from GCST009019; TREM2 from GCST005549; TTR from GCST007319; FUS from GCST007320; TOMM40 from GCST000682; BIN1 from GCST005922; and ACE from GCST90013835.

Second, for the rest of the 32 genes that do not exist in the GWAS Catalog, 4 of them are included in the known database. In detail, PSEN1 and PSEN2 are both in UniProtKB and MedlinePlus, while SNCA and CSK3B are in UniProtKB. Eventually, for the 28 genes that are not reported by GWAS or known databases, 23 of them are suggested to be AD-related with confirmed phenotype description.

**Fig. 5:**
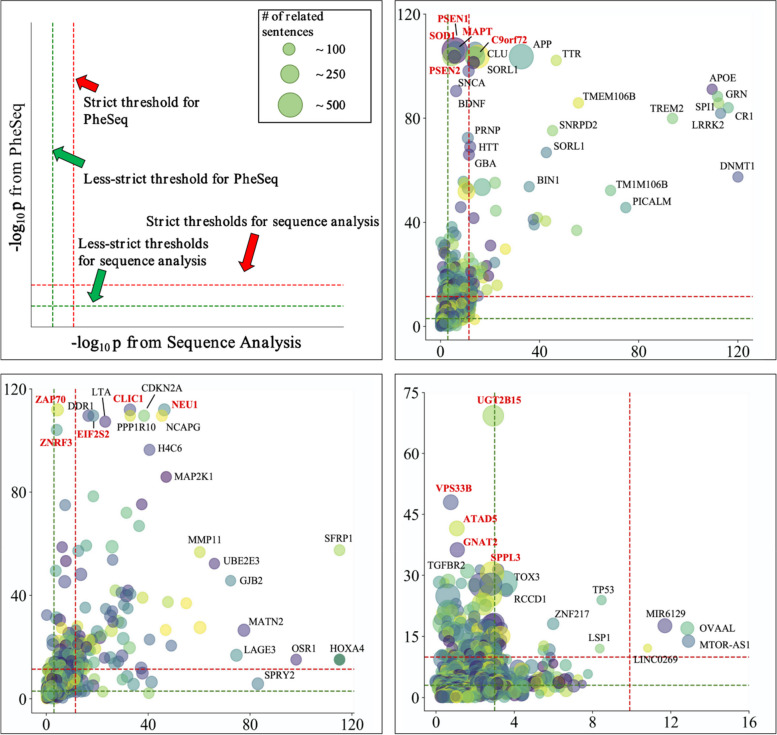
The $-\log p$ plots of overlapping and recalled genes after applying PheSeq and sequence analysis in AD, BC, and LC. **a** Layout of the $-\log p$ plot. The *x*-axis and *y*-axis denote the $-\log$ *p* value from the sequence analysis and the PheSeq model respectively. The red line refers to a strict threshold line such as Benjamini FDR in our case, and the green line refers to a less strict threshold line such as $-\log 0.005$ in our case. Genes are labeled when overlapped in PheSeq and sequence analysis or recalled by PheSeq. **b** The $-\log p$ plot of significance for both PheSeq and sequence analysis in AD. Five genes are marked in red, i.e., MAPT, PSEN1, C9orf72, SOD1, and PSEN2. All of them are PheSeq recalled genes, which obtain high significance in PheSeq but obtain less or limited significance in GWAS. **c** The $-\log p$ plot in BC. Five PheSeq recalled genes are chosen and marked in red, i.e., NEU1, ZAP70, EIF2S2, ZNRF3, and CLIC11. These genes obtain comparatively higher significance in PheSeq than that in sequence analysis. **d** The $-\log p$ plot in LC. The five marked genes are UGT2B15, VPS33B, ATAD5, GNAT2, and SPPL3. All five genes show strong significance in PheSeq but limited significance in sequence analysis

To compare the global prioritization results between PheSeq and sequence analysis, a cumulative chart for database hits for the top 50 filtered genes is given in Fig. 5b.

Overall, the results suggest that the PheSeq model effectively leverages the synergy among heterogeneous association data, alleviating the limitations of using single-source association significance data.

### Genes with significance in PheSeq and single-omics sequence analysis: a comparative observation

It is noted that the sequence analysis used in comparative experiments may introduce errors, particularly when considering the inherent instability of results obtained from single-omics sequence analyses. Consequently, the sequence analysis unavoidably overlooks certain known significant associations and may erroneously produce false positive results. PheSeq, on the other hand, aims to reduce the error by data fusion. Therefore, a comparative analysis is performed for both types of experiments.

To display and further investigate each overlapping and recalled gene by considering its significance value both on PheSeq and the sequence analysis, $-\log p$ plots for all significant genes in AD, BC, and LC are given in Fig. 5. In this figure, the horizontal $-\log p$ axis refers to the association significance obtained by sequence analysis, while the vertical $-\log p$ axis corresponds to the generated *p*-value by PheSeq. In addition, the size of the circle for each gene reflects the count of the phenotype descriptions related to the gene (Fig. 5a).

Intuitively, these figures offer a means by which to investigate the genes with overlapped significance both in sequence analysis and phenotype description. In particular, the plot is separated into sections by threshold lines. The genes with overlapped significance genes are located in the top right corner of the plot, which pass the significance test in sequence analysis and in the meantime carry sufficient association semantics. Meanwhile, the newly reported significant genes by PheSeq are located in the top left section of the plot, which may show less or limited significance in sequence analysis.

In the context of AD, genes with overlapping associations, including APOE, GRN, LRRK2, and SPI1, are visually presented in the top right corner (Fig. 5b), all of which pass the significance test in GWAS and possess sufficient AD-relevance association semantic by PheSeq. Furthermore, genes with less significance in the sequence analysis, e.g., PSEN1, SOD1, MAPT, C9orf72, and PSEN2, are displayed in the top left section. Among the four, PSEN1 and PSEN2 are known AD-related genes, reported in AlzGene [67], while C9orf72 and SOD1 are known to be relevant to neurogenetic disease and possess AD-relevant literature support in GeneCards [68].

In BC, overlapped genes, such as SFRP1, HOXA4, and OSR1 genes, are clearly displayed (Fig. 5c). The location of these genes in the figures show that these genes possess both significance in sequence analysis and PheSeq. Again, we are focusing on the recalled genes by PheSeq. Here, PheSeq recalled less significant genes in sequence analysis such as NEU1, ZAP70, EIF2S2, ZNRF3, and CLIC1, while all of which possess strong significance in PheSeq. The literature review as well shows the relevance of BC to these genes, e.g., NEU1 [69], ZAP70 [70], EIF2S2 [71], ZNRF3 [72], and CLIC1 [73].

Similar observations are carried on for LC, where overlapped genes such as MIR6129, OVAAL, MTOR-AS1, and LINC0269 are displayed in the top right corner of Fig. 5d. Meanwhile, the top left part of the figure indicates PheSeq-recalled genes. The literature review again shows the relevance of LC to these genes, e.g., UGT2B15 [74], VPS33B [75], ATAD5 [76], GNAT2 [68], and SPPL3 [77].

In conclusion, association study results obtained by PheSeq and a single sequence analysis can be simultaneously observed by the above figures. In addition, this figure enables specific investigation on overlapped genes or newly recalled genes by PheSeq after data fusion. The analysis in the three cases suggests that the genes recalled by Pheseq may not be directly associated with the target disease, but they with a high chance exhibit relevance via database or literature review.

### Impact of phenotype description on PheSeq with association interpretability

PheSeq incorporates rich semantic information within its data fusion framework, and it leverages the synergy between the sequence analysis and association descriptions. As a result, PheSeq retrieves a vast dataset of phenotype sentences and bio-concepts for interpreting the prioritized gene-disease association. In summary, 14,084 phenotype sentences are utilized by PheSeq to support 1024 prioritized genes in AD. With an average of 13 phenotype sentences per gene, this dataset includes 1849 GO terms and 1351 HPO terms. In BC and LC, 2250 and 10,440 phenotype sentences are obtained, respectively, with each gene associated with an average of 9 and 10 phenotype sentences. More details on the validation of embedding quality, and the statistic of phenotype description are provided in Additional file 3.

Actually, the PheSeq model prioritizes the gene-disease associations by perceiving corresponding description descriptions. As per observation, genes recalled by PheSeq generally possess pertinent phenotype descriptions. Taking MAPT in AD as an example, it is known to be relevant to the etiology of AD by the widely accepted Tau protein hypothesis, although it fails to pass the significance test in GWAS. As can be observed from Table 3, the most frequently cited phenotype descriptions related to MAPT include “Neurofibrillary tangles” (HP:0002185), “Hyperphosphorylation” (GO:0048151), “Cognitive impairment” (0100543), “Microtubule binding” (GO:0008017), “Long-term synaptic potentiation” (GO:0060291), and “Microtubule polymerization potentiation” (GO:0046784). According to the Tau protein hypothesis, hyperphosphorylation of the Tau protein leads to its aggregation, ultimately disrupting microtubule stability and resulting in the formation of neurofibrillary tangles―a hallmark pathological feature of AD. The observation shows that the top ranked associated phenotype descriptions are highly relevant and supportive for the MAPT-AD association.

**Table 3:** Associated phenotypes for PheSeq recalled genes

Gene	Significant in PheSeq	Significant in SA^a^	# of phenotype	Phenotype ID	Phenotype term	# of citation
Alzheimer’s disease						
MAPT	Strict	Less	118	HP:0002185	Neurofibrillary tangles	202
				GO:0048151	Hyperphosphorylation	105
				HP:0100543	Cognitive impairment	69
				GO:0008017	Microtubule binding	24
				GO:0060291	Long-term synaptic potentiation	21
				GO:0046785	Microtubule polymerization potentiation	15
PSEN1	Strict	Less	121	HP:0011034	Amyloidosis	102
				GO:0016310	Phosphorylation	55
				GO:0016236	Macroautophagy	51
				GO:0060291	Long-term synaptic potentiation	32
				HP:0002185	Neurofibrillary tangles	21
				HP:0100256	Senile plaques	19
C9orf72	Strict	Limited	128	GO:0008384	IkappaB kinase activity	26
				GO:0004784	Superoxide dismutase activity	17
				GO:0003777	Microtubule motor activity	14
				HP:0410170	Hippocampal atrophy	13
				HP:0007112	Temporal cortical atrophy	11
				HP:0002354	Memory impairment	6
SOD1	Strict	Less	84	GO:0004784	Superoxide dismutase activity	153
				HP:0007373	Motor neuron atrophy	48
				HP:0003287	Abnormality of mitochondrial metabolism	20
				GO:0000422	Autophagy of mitochondria	19
				GO:0003777	Microtubule motor activity	15
PSEN2	Strict	Less	84	GO:0042982	Amyloid precursor protein metabolic process	88
				GO:0006954	Inflammatory response	78
				HP:0100256	Senile plaques	73
				GO:0060291	Long-term synaptic potentiation	65
				HP:0002529	Neuronal loss in central nervous system	32
Breast cancer						
NEU1	Strict	Less	67	GO:0001525	Angiogenesis	87
				GO:0001837	Epithelial to mesenchymal transition	77
				GO:0006915	Apoptotic process	43
				GO:0007155	Cell adhesion	26
				GO:0012501	Programmed cell death	24
ZAP70	Strict	Limited	74	GO:0001816	Cytokine production	146
				GO:0002870	T cell anergy	78
				GO:0012501	Programmed cell death	43
				GO:0008283	Cell population proliferation	24
				GO:0042110	T cell activation	24
EIF2S2	Strict	Less	34	GO:0004030	Aldehyde dehydrogenase [NAD(P)+] activity	155
				GO:0007049	Cell cycle	112
				GO:0042571	Immunoglobulin complex, circulating	56
				GO:0019815	B Cell receptor complex	46
				GO:0005850	Eukaryotic translation initiation factor 2 complex	43
ZNRF3	Strict	Limited	32	GO:0001837	Epithelial to mesenchymal transition	96
				GO:0004693	Cyclin-dependent protein serine/threonine kinase activity	38
				GO:0016055	Wnt signaling pathway	37
				GO:0044214	Spanning component of plasma membrane	32
				GO:0008283	Cell population proliferation	12
CLIC1	Strict	Less	57	GO:0005161	Platelet-derived growth factor receptor binding	53
				GO:0001837	Epithelial to mesenchymal transition	38
				GO:0005006	Epidermal growth factor-activated receptor activity	51
				GO:0005104	Fibroblast growth factor receptor binding	37
				GO:0005172	Vascular endothelial growth factor receptor binding	31
Lung Cancer						
UGT2B15	Strict	Limited	63	GO:0004707	MAP kinase activity	155
				GO:0005041	Low-density lipoprotein particle receptor activity	91
				GO:0005007	Fibroblast growth factor-activated receptor activity	82
				GO:0005104	Fibroblast growth factor receptor binding	82
				GO:0005172	Vascular endothelial growth factor receptor binding	65
VPS33B	Strict	Limited	30	GO:0001837	Epithelial to mesenchymal transition	147
				GO:0005006	Epidermal growth factor-activated receptor activity	51
				GO:0016192	Vesicle-mediated transport	42
				HP:0100630	Nasopharyngeal	39
				GO:0005092	GDP-dissociation inhibitor activity	12
ATAD5	Strict	Limited	29	GO:0072671	Mito-associated ubiquitin-dependent protein catabolic process	50
				GO:0005007	Fibroblast growth factor-activated receptor activity	47
				GO:0005011	Macrophage colony-stimulating factor receptor activity	45
				GO:0008384	IkappaB kinase activity	31
				GO:0033868	Goodpasture-antigen-binding protein kinase activity	21
GNAT2	Strict	Limited	28	GO:0005680	Anaphase-promoting complex	111
				GO:0001532	Interleukin-21 receptor activity	67
				GO:0033868	Goodpasture-antigen-binding protein kinase activity	63
				GO:0072671	Mito-associated ubiquitin-dependent protein catabolic process	54
				GO:0004709	MAP kinase activity tangles	21
SPPL3	Strict	Limited	28	GO:0001837	Epithelial to mesenchymal transition	76
				GO:0005104	Fibroblast growth factor receptor binding	65
				GO:0005149	Interleukin-1 receptor binding	32
				GO:0005160	Transforming growth factor beta receptor binding	26
				GO:0005164	Tumor necrosis factor receptor binding	12

^a^*SA *Sequence analysis

We further investigated four such genes, namely PSEN1, c9orf72, SOD1, and PSEN2, all of which displayed robust significance in PheSeq, despite exhibiting less or limited significance in sequence analysis.

Table 3 presents examples and statistics of the phenotype descriptions including bio-concepts and sentences. Except for C9orf72, the rest of them are all recalled ones by PheSeq. Here, frequently mentioned bio-concepts include “Senile plaques” (HP:0100256), “Neurofibrillary tangles” (HP:0002185), “Hippocampal atrophy” (HP:0410170), “Abnormality of mitochondrial metabolism” (HP:0003287), and “Inflammatory response” (GO: 0006954). These phenotype descriptions are known to be relevant to AD, thus suggesting a potential gene list for further AD-gene association investigations.

Similarly, an inquiry is undertaken regarding NEU1, ZAP70, EIF2S2, ZNRF3, and CLIC1 in BC. Remarkably, these genes exhibit significant importance in PheSeq analysis, despite showing relatively modest significance in sequence analysis.

In accordance with the aforementioned observations in the AD case, phenotype descriptions with high association relevance are derived. Specifically, bio-concepts such as “Angiogenesis” (GO:00001525), “Cytokine production” (GO:0001816), “Epidermal growth factor-activated receptor activity” (GO:0005006), “Aldehyde dehydrogenase [NAD(P)+] activity” (GO:0004030), and “Wnt signaling pathway” (GO:0016055) are frequently mentioned.

Meanwhile, UGT2B15, VPS33B, ATAD5, GNAT2, and SPPL3 exhibit a significant impact on PheSeq in LC and win corresponding literature support [68, 74–77], despite not meeting the reference threshold in sequence analysis. Consistent with previous observations in AD and BC cases, these genes are commonly associated with LC-relevant phenotypes, including “Low-density lipoprotein particle receptor activity” (GO:0005041), “Fibroblast growth factor-activated receptor activity” (GO:0005007), “GDP-dissociation inhibitor activity” (GO:0005092), “Goodpasture-antigen-binding protein kinase activity” (GO:0033868), and “Transforming growth factor beta receptor binding” (GO:0005160).

In summary, these results indicate that PheSeq underscores the disease-specific phenotype descriptions and incorporate them with sequence analysis significance. Remarkably, PheSeq holds particular importance in situations where a single sequence analysis may elicit systematic bias and flawed predictions of crucial genes. In such instances, PheSeq serves as an effective tool for establishing a connection between phenotype descriptions and association significance in sequence analysis and helps to recall the significant genes.

### Impact of prior knowledge on PheSeq with association prediction: an ablation study

In the aforementioned analysis, we compare the performance of PheSeq with that of a single sequence analysis in three distinct case studies. It is essential to note that, as a data fusion method, PheSeq inherently incorporates prior knowledge from literature and networks. Consequently, PheSeq is a model integrating prior knowledge and holds an inherent advantage over conventional sequence analysis models. In this section, we conduct an ablation study to evaluate how prior knowledge is incorporated into the PheSeq model. We systematically remove specific prior information and rerun the entire prediction process to assess the impact accordingly.

Based on the publication dates of omics data, we exclude all literature data beyond those time points. Specifically, for AD, the literature cutoff date is set at October 27, 2013. Correspondingly, for BC and LC, the respective dates are January 28, 2016. Consequently, this approach results in a significant compression of the prior knowledge derived from the literature. In the original experiments, the literature on AD covers 14,261 genes; however, with the cutoff set on October 27, 2013, only 1017 genes are now covered. In the case of BC, the gene coverage decreases from 10,498 to 3,399, and in LC, the reduction rate is greater, dropping from 20,460 to 749 genes.

PheSeq in the ablation setting predicts 391 significant genes associated with AD, 1398 significant genes associated with BC, and 172 ones with LC. Despite the relatively limited inclusion of prior literature knowledge for these genes, the results in Table 4 clearly demonstrate two patterns. First, predicted significant genes typically carry a higher proportion of literature knowledge. For instance, among the 391 key AD genes, each gene, on average, possesses 21.17 literature references, 31.80 pieces of related sentence evidence, and 11.54 core concepts, whereas in the corresponding non-significant genes, these values are only 2.32, 3.28, and 2.90, respectively. Second, due to the preservation of PPI data in prior knowledge, prioritized genes are more likely to be adjacent to other significant ones. For instance, among the 391 AD significant genes, statistical analysis of information from their top 10 neighbors reveals an average of 5.54 significant genes per gene, with a cumulative literature count of 115.35, a sentence evidence count of 173.22, and a concept count of 62.86. In contrast, for non-significant genes, the number of significant genes among their top 10 neighbors decreases to 3.13, with corresponding literature, sentence, and concept counts of 3.08, 4.36, and 3.87, respectively. The two patterns are observed as well in BC and LC case studies.

**Table 4:** Investigation of prior knowledge derived in the significant or non-significant genes in the ablation study

	Gene count	Aveg. literature	Aveg. sentence	Aveg. concept	# sign. neighbors	# literature	# sentence	# concept
Alzheimers’ disease								
Sign. genes	391	21.17	31.80	11.54	5.54	115.35	173.22	62.86
Non-sign. genes	626	2.32	3.28	2.90	3.13	3.08	4.36	3.87
Breast cancer (BC)								
Sign. genes	1398	13.96	21.67	13.65	6.51	80.59	125.84	79.04
Non-sign. genes	2001	2.36	3.29	3.63	5.19	4.33	6.03	6.65
Lung cancer (LC)								
Sign. genes	172	17.23	22.29	12.12	4.78	88.90	115.00	61.16
Non-sign. genes	577	1.56	2.03	2.29	2.94	0.41	0.54	0.62

In short, significant genes exhibit extensive prior knowledge, either encompassing abundant literature in historical data or demonstrating strong associations with significant disease-related genes in PPI networks.

Taking PICALM as an example, this gene is notably associated with a substantial amount of AD literature. As of the end of 2023, a total of 264 publications are available for PICALM, with 112 publications retained before the cutoff in 2013. This abundance of literature contributes to PICALM being identified as a significant gene with a high probability in the ablation study conducted by PheSeq. Similarly, ESR1 in LC also maintains a considerable literature count, totaling 132 publications by the end of 2023 and retaining 54 publications before the cutoff in the preceding years of 2016.

In AD, GBA emerges as the gene exhibiting the strongest association in the PPI network. Its neighbors, such as UGCG, PSAP, GALC, and SGMS2, are all linked to known AD pathological processes and exhibit significant *p*-values in sequence analysis, namely 0.045, 0.029, 0.00056, and 0.0014, respectively. This significantly increases the likelihood of PheSeq identifying GBA as a significant gene.

Similarly, in BC, the NEU1 gene is strongly linked to several significant genes in the PPI network, including GLB1 (4.11e−11), ARSA (5.15e−05), and GAL3ST1 (1.99e−13). This, in turn, leads to PheSeq maintaining positive predictions for these genes in the ablation study.

In summary, the observed patterns in the ablation experiments indicate that despite the extensive removal of literature prior knowledge, the predicted significant genes still predominantly retain both literature and network priors. This in turn aligns with the initial purpose of data fusion.

**Fig. 6:**
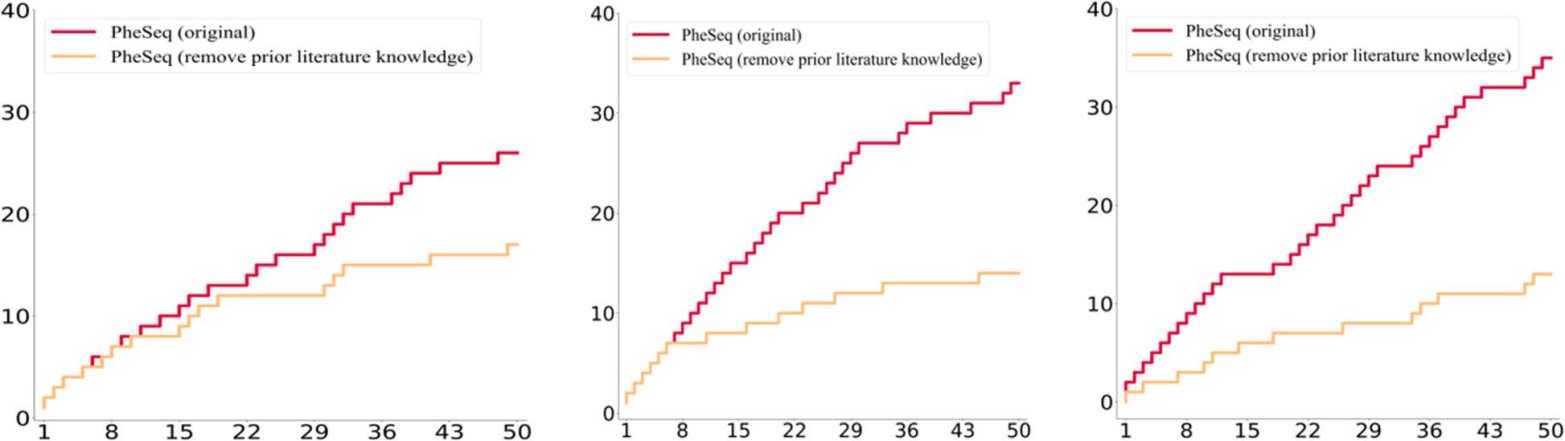
The top 50 significant genes with cumulative charts in DISEASES in the ablation study

Furthermore, we evaluate the predictive capacity of PheSeq with removed prior literature knowledge, and the top 50 significant genes with the cumulative charts in DISEASES are shown in Fig. 6a. The yellow line represents the ablation method where literature priors are excluded, while the red line corresponds to the original PheSeq method. In the cumulative line plot, it is observable that the yellow line consistently remains below the red line. This result indicates a significant decline in the predictive capability of PheSeq when a substantial amount of literature priors is removed, and it aligns with the data fusion concept in PheSeq.

### Comparison of other data fusion models

**Fig. 7:**
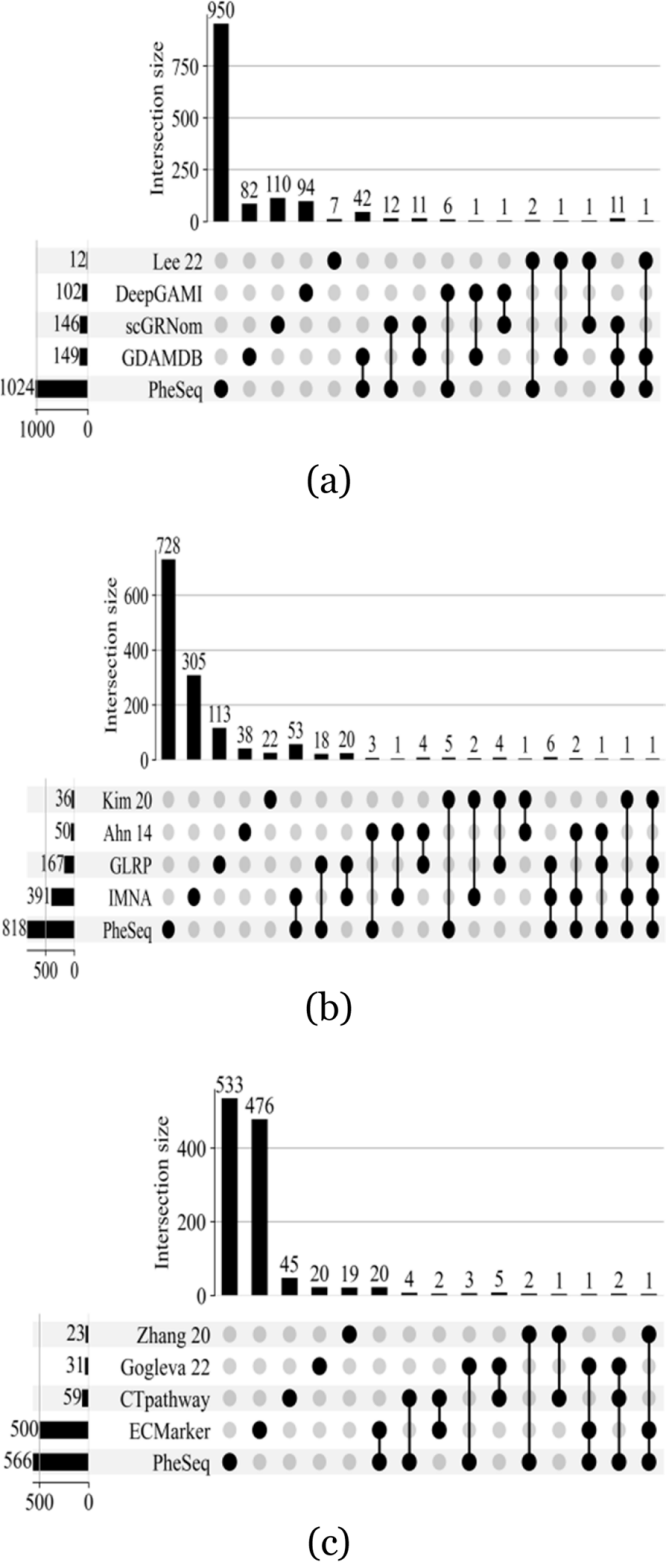
Overlap of significant genes from different data fusion methods on gene-disease associations. **a** AD. **b** BC. **c** LC

As a representative data fusion algorithm, PheSeq combines two distinct types of association information: sequence analysis data and embedding data. When addressing gene-disease associations, there are diverse strategies for data incorporation and model selection within data fusion algorithms. Even when examining the same disease, variations in results among different fusion methods can arise due to the use of diverse data modalities. Figure 7 illustrates the overlap of significant genes under various methodologies. As depicted in the figure, achieving a high degree of overlap between different methods is challenging, regardless of the number of significant genes predicted by each approach.

Nevertheless, conducting a comparative analysis of outcomes from various data fusion methods, including PheSeq, remains crucial for obtaining a comprehensive evaluation of PheSeq’s performance. As shown in Table 5, different methods cover various data modalities, including data from GWAS, gene expression, gene regulatory network (GRN), expression quantitative trait loci (eQTL) high-throughput chromosome conformation capture (Hi-C), copy number alteration (CNA), literature, and protein-protein interaction. The number of significant genes varies across methods, with Lee et al. [78] having the lowest at 12 and PheSeq having the highest at 1024 in AD. In BC, Kim et al. [79] report the lowest count at 35 while PheSeq has the highest count at 818. In LC, Zhang et al. [80] have the lowest at 23 whereas PheSeq exhibits the highest at 566. This likely reflects differences in the identification of significant genes when using different methods and data modalities.

**Table 5:** Comparison of different data fusion methods on gene-disease associations

Method	Data modality	Strategy^a^	Interpretation	Count^b^	Hit^c^
Alzheimers’ disease					
Lee 22 [78]	GWAS + eQTL	Early	/	12	8
DeepGAMI [81]	Gene expression + GRN +eQTL	Early	Enrichment analysis	102	2
scGRNom [82]	GWAS + Hi-C	Intermediate	Enrichment analysis	146	43
GDAMDB [32]	GWAS $+$ literature	Late	/	149	72
PheSeq	GWAS $+$ literature $+$ PPI	Late	Phenotype and literature	1024	151
Breast cancer					
Kim 20 [79]	CNA + gene expression + methylations + clinical info	Intermediate	/	36	5
Ahn 14 [83]	Transcriptome + pathway	Early	/	50	2
GLRP [84]	Gene expression + PPI	Early	Interpretable neural network + pathway analysis	167	5
IMNA [85]	GWAS + eQTL	Early	Literature	391	24
PheSeq	Transcriptome $+$ literature $+$ PPI	Late	Phenotype and literature	818	159
Lung cancer					
Zhang 20 [80]	Methylation + gene expression	Early	/	23	2
Gogleva 22 [86]	CRISPR + knowledge graph + literature	Intermediate	Interpretable recommendation system	31	8
CTpathway [87]	Gene expression + transcriptome + pathway + PPI	Intermediate	Pathway analysis	59	12
ECMarker [88]	Gene expression + clinical phenotype	Early	Interpretable neural network	500	19
PheSeq	Methylation $+$ literature $+$ PPI	Late	Phenotype and literature	566	342

^a^Data fusion strategy. Early for data-level, intermediate for joint-level, and late for decision-level

^b^Count of significant genes

^c^Hits of significant genes in DISEASES

There are three main types of data fusion strategies used in machine learning; early (data-level), intermediate (joint-level), and late (decision-level) [89, 90]. In the early data fusion algorithms, data from various sources, once fully collected, are mapped to a unified data space through vectorization methods such as concatenation or addition. Subsequently, a machine learning model is employed for knowledge-based decision-making. Researches [78, 80, 81, 83–85, 88] fall into this scope. In contrast, intermediate data fusion algorithms often utilize a series of models within a step-wise set, where different models handle distinct stages of data, ultimately completing data fusion and knowledge-based decision-making within a single pipeline. This type of algorithm includes researches [79, 82, 86, 87]. Late data fusion algorithms, on the other hand, involve the simultaneous processing of data from different sources by various models, achieving integrated decision-making. Although the selected comparative experiments only represent a small portion of the data fusion methods for three case studies, it is suggested that early and intermediate data fusion methods remain predominant, and late data fusion methods are relatively less frequently employed. GDAMDB [32] and PheSeq stand as representatives of late data fusion methods, utilizing Bayesian networks to learn the distribution relationships among data variables, offering interpretable fusion decisions.

In addition, the interpretation approaches vary widely among these methods. While some methods rely on enrichment analysis and pathway analysis, others incorporate more sophisticated techniques such as interpretable neural networks or recommendation systems. Additionally, some methods do not explicitly specify their interpretation approach. This diversity highlights the complexity of interpreting integrated data and underscores the need for tailored approaches based on the specific objectives of each study.

Finally, we utilize DISEASES as the external dataset referenced to compare the performance of predictive capacity among these methods. As detailed in the rightmost column of the table, PheSeq exhibits superior predictive performance in BC and LC, outperforming other methods in precision and recall. For instance, PheSeq recalls 159 DISEASES genes out of 818 predicted significant genes. Both the amount and the ratio are greater than the rest methods. In AD, while PheSeq recalls 151 DISEASES genes, this is attributed to its larger overall prediction quantity. Conversely, Lee et al. [78] and GDAMDB [32] demonstrate higher precision, with GDAMDB displaying notably high recall values. This also underscores the advantages of the late data fusion approach.

In summary, PheSeq stands out as a late data fusion algorithm in the context of gene-disease associations, predominantly employing phenotype descriptions extracted from literature to enhance the interpretive aspects of the obtained results.

### Association interpretation in a visualized phenotype description network

Benefiting from the good amount of phenotype description and sentence support, we derive abundant phenotype descriptions for gene-disease associations. To summarize all the PheSeq-prioritized genes with the collected bio-concepts and sentences, a visualized phenotype description network is built for AD, BC, and LC, separately. In the network, the significant genes (both from PheSeq and sequence analysis) and the bio-concepts are treated as nodes, and a gene-concept edge is linked when a sentence description addressing the association is available. The network is released in a user-friendly webpage^1^, while the pipeline of the network construction is introduced in Additional file 3.

The network offers diverse patterns of association interpretations that serve to enhance the comprehension of the mechanisms that underlie gene-disease associations.

#### Pattern 1. GO enrichment analysis

**Fig. 8:**
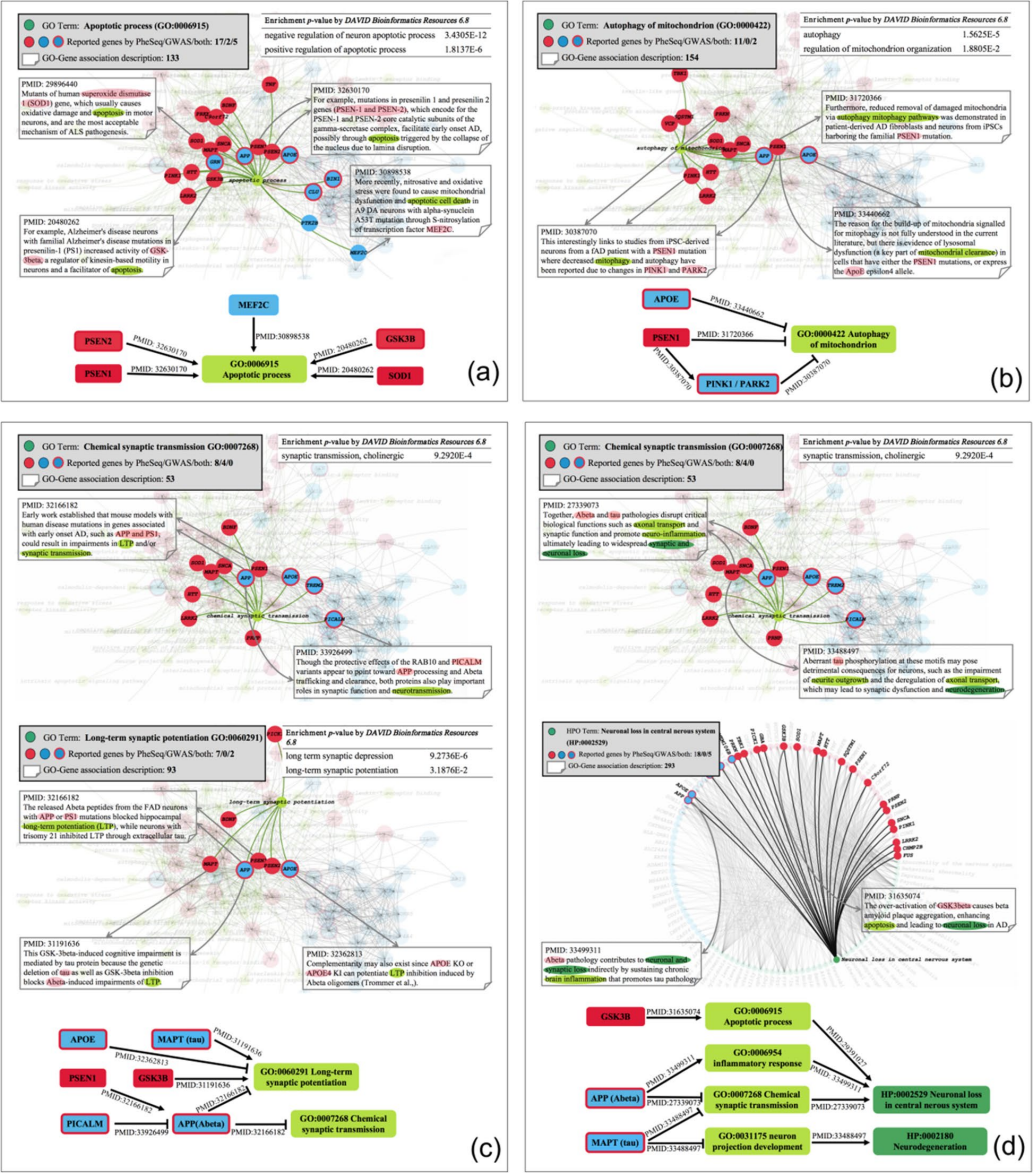
Association interpretation in the visualized phenotype description network for AD. **a** Observation of the gene-phenotype links. There are 19 significant PheSeq genes linked with the phenotype term “apoptosis process” in AD, and sequence analysis provides 7 significant links. **b** Gene-gene interaction through genes with shared phenotype descriptions. APOE and PSEN1 inhibit the autophagy of mitochondria directly, as reported in PMID:33440662 [95] and PMID:31720366 [96]. Meanwhile, PSNE1 inhibits this biological process by affecting PINK1 and PARK2. **c** Multiple GO terms lead to complex gene pathological pathways. PSEN1 and GSK3B are exclusively found in PheSeq, while the other four, i.e., APOE, MAPT, APP, and PICALM are both found by PheSeq and IGAP GWAS. All of them interact with each other and lead to two biological processes, long-term synaptic potentiation, and synaptic transmission. **d** Links between genes and GO or HPO interpret a multi-level pathology mechanism. By tracing two HPO terms, i.e., neuronal loss in the central nervous system and neurodegeneration, to GO terms and their linked genes, multi-level links are formulated. Three genes are included in these links, in which GSK3B is exclusively found by PheSeq, while APP and MAPT are separately found by PheSeq and IGAP GWAS. These links unveil a cascade mechanism that starts from gene involvement in multiple biological processes and ends in two phenotypic processes

The network enables GO enrichment analysis. Here, four gene sets are shown in Fig. 8a, b, c with GO terms corresponding to apoptosis [91], mitophagy [92], chemical synaptic transmission [93], and long-term synaptic potentiation [94]. In Fig. 8a, 24 genes are linked with the “Apoptotic process” (GO:0006915), supported by 133 pieces of sentence evidence. In total, the 24 genes consist of 7 ones from GWAS, 17 ones from PheSeq, and 5 ones that overlapped. After applying the hypergeometric test, the gene set is significantly enriched in “Negative regulation of neuron apoptotic process” (GO:0043524) with an association significance of 3.4305E-12 and “Positive regulation of apoptotic process” (GO:0043065) with an association significance of 1.8137E−06. The results confirm the relevance of these 24 genes to the “apoptosis process.” Moreover, all the GO-linked genes in Fig. 8b,c pass the corresponding GO enrichment test.

#### Pattern 2. Link genes from two sources

The inclusion of significant genes identified by both PheSeq and sequence analysis provides avenues for further investigation into pathological mechanisms. Taking the five genes in Fig. 8a as an example, MEF2C is a GWAS-reported gene, the S-nitrosylation of which causes mitochondrial dysfunction and apoptotic cell death in neurons. Furthermore, PheSeq prioritized genes such as PSEN1, PSEN2, SOD1, and GSK3B are also added to the linking graph. Among them, PSEN1 and PSEN2 are known AD-related genes, the mutation of which contributes to the clinical syndrome of early-onset AD (EOAD) through apoptosis. In addition, SOD1 and GSK3B both trigger apoptosis in neurons. Evidence in literature (PMID: 32006534 [97]) indicates that mutations in PSEN1 increase the activity of GSK3B, cause apoptosis, and facilitate AD. These observations imply that all the linked genes are related to apoptosis and AD pathology.

#### Pattern 3. Hybrid gene-phenotype associations

The network facilitates hybrid investigation of gene-phenotype associations. First, exploring genes that share phenotype descriptions holds the potential to reveal gene-gene interactions. As shown in Fig. 8b, APOE and PSEN1 inhibit the autophagy of mitochondria directly, as reported in PMID:33440662 [95] and PMID:31720366 [96]. Meanwhile, PSNE1 inhibits this biological process by affecting PINK1 and PARK2. In detail, PMID:31720366 [96] claims that PSEN1 mutation reduces the removal of damaged mitochondria via autophagy mitophagy pathways, PMID:30387070 [98] claims that patients with a PSEN1 mutation where there is decreased mitophagy and autophagy have been reported due to changes in PINK1 and PARK2, and PMID:33440662 [95] figures out the PSEN1 mutations or expresses the APOE which induces the lysosomal dysfunction, which is a key part of the mitochondrial clearance. After combining the three pieces of evidence, the interaction between these genes is inferred. Mutations in the PSEN1 induce changes in PINK and PARK2, which induces lysosomal dysfunction, thus causing mitochondrial accumulation by inhibiting mitophagy in iPSC-derived neurons of AD patients.

Second, an integrative analysis of multiple GO terms leads to the discovery of complex gene pathological pathways. For instance, sentence evidence from PMID:32166182 [99], 33926499 [100], 31191636 [101], and 32362813 [102] in Fig. 8c curates the pathway information: the released Abeta peptides from the FAS neurons with APP or PSEN1 mutations causes synaptic inhibition, such as long-term potentiation (LTP) blockade and neurotransmission defects. Meanwhile, the Abeta protein encoded by APP plays a crucial role in this pathway. In more detail, APOE potentiates LTP inhibition induced by Abeta oligomers. In addition, the genetic deletion of tau protein, as well as GSK3B inhibition, blocks Abeta-induced impairments of LTP. Eventually, PICALM variants appear to cause Abeta trafficking and clearance, thereby protecting the synaptic function and neurotransmission.

Third, exploring links between genes and GO or HPO can enhance the understanding of a comprehensive pathology mechanism across multiple levels. As shown in Fig. 8d, GSK3B, APP, and MAPT induce two clinical phenotypes in AD pathology, neuronal loss, and neurodegeneration, by affecting four molecular-level physiological processes, including the apoptotic process, inflammatory response, synaptic transmission, and neuron projection development.

Finally, an evidence-supported gene-GO network contributes to integrating the findings, pinpointing vital disease-associated genes. For instance, PSEN1 is linked with a good variety of GO terms, which make the PSEN1-centric gene-GO links illuminative. Figure 8a, b ,c shows that PSEN1 facilitates early-onset AD possibly through apoptosis triggered by the collapse of the nucleus due to lamina disruption. In addition, PSEN1 reduces the removal of damaged mitochondria via autophagy mitophagy pathways in patient-derived AD fibroblasts and neurons from iPSCs. Furthermore, PSEN1 mutation blocks hippocampal LTP by promoting the release of Abeta peptides in FAD neurons. Altogether, the network is beneficial for a comprehensive understanding of the different mechanisms that PSEN1 plays in the AD process.

Interestingly, although GSK3B has not been reported to be AD-related, Fig. 8c, d shows that it is involved in two AD pathological pathways. One is the increased activity of GSK3B induced by PSEN1, followed by facilitated apoptosis. The other is the genetic deletion of tau protein mediates the inhibition of GSK3B, thereby blocking Abeta-induced impairments of LTP.

#### Pattern 4. Association augments with auxiliary PPI info

Considering the rich PPI information encompassed within the data modalities integrated by PheSeq, we also incorporate the representation of PPI connections in the visualized phenotype description network. The black edges between genes in the network represent PPI information sourced from the STRING database. Previous experiments demonstrate that, in the absence of literature data, PPI links crucially contribute to graph embedding, aiding PheSeq in retrieving relevant significant genes. Additional file 3 provides examples of the hub or common gene nodes in the PPI network, which link to other significant gene neighbors in PPI connections. Examples suggest that the observation of auxiliary PPI attributes provides augmented mechanistic insights for a given gene-disease association.

In short, the visualized phenotype description network contributes to addressing gene association in an interpretable manner and provides further potential to unveil the disease pathology mechanism.

## Discussion

In the present day, the co-existence of sequence analysis outcomes and textual resources has emerged as an increasingly pervasive practice. In light of this trend, data fusion of the above heterogeneous data holds considerable promise for advancing comprehensive data fusion techniques.

The scenario focused on in this research is such a case when a rich resource of *p*-values and descriptive texts are available, both of which form a pair of heterogeneous association datasets supporting the discovery of the gene-disease associations.

The PheSeq model effectively integrates the advantages of two types of data by leveraging the heterogeneous synergy in a Bayesian deep learning framework. PheSeq specifically utilizes the DL perception module to generate high-quality embedding representations from phenotype descriptions. Additionally, it makes use of the Bayesian network to effectively model the uncertainty of observation and infer the inherent dependence relations among gene-disease associations.

PheSeq takes advantage of the interpretability nature of the phenotype descriptions. The use of bio-concepts and sentence evidence further improves the interpretability of PheSeq results. Moreover, the knowledge inference patterns shown in Fig. 8 suggest that only when literature and sequence information are well integrated can the model unveil hidden in-depth mechanisms out of the network.

In addition to a promising data fusion idea, PheSeq also encourages certain concerns for further exploration of gene-disease associations.

First, PheSeq does not functionas a predictive algorithm solely focused on achieving absolute confidence in association prioritization. Instead, our primary objective is to addressthe inherent limitations of inference derived from single-omics sequence analysis. Therefore, we adopt a data fusion approach to facilitate interpretable novel associations.

Second, the prioritized gene-disease association needs to be investigated with a methodical approach. As evident in sequence analysis, depending solely on statistical significance and employing stringent cutoff criteria may result in high false negatives. As shown in Fig. 5, the prioritization of PheSeq does not always align with the sequence analysis. The significant *p*-value may be discarded due to missing embedding support, and the non-significant *p*-value may be recalled due to supportive embedding. Fortunately, the PheSeq model provides strong evidence traceability, which enables further validation or investigations of genes of concern by checking the evidence support, even if the gene has lower rankings.

Third, considerations are needed when applying PheSeq in a general genotype-phenotype association study. For example, the appropriate thresholding strategy is needed to evaluate the significance of associations after the sequence analysis. In addition, appropriate benchmarks datasets, such as DISEASES used in our cases, are used for the sake of evaluation. Furthermore, the inconsistency of association significance and phenotype description needs to be investigated ahead of the model implementation.

## Conclusion

In conclusion, this research performs a worth-trying attempt in heterogeneous association data fusion This framework successfully bridges the phenotype description perception and *p*-value uncertainty inference. The association significance is utilized as a fine-grained weak signal for the association significance. Overall, it is an inspiring idea to unveil genotype-phenotype associations and investigate the potential relation dependency through data perception, data fusion, and probabilistic inference in a novel Bayesian framework.

### Supplementary Information

**Additional file 1.** Usage Guideline of PheSeq Code. This file provides detailed parameter descriptions and command line usage instructions for all scripts involved in the PheSeq.

**Additional file 2.** Model Solution and Implementation. This file contains comprehensive algorithmic solutions implemented in PheSeq, detailing the models and their implementations.

**Additional file 3.** Phenotype Description by PheSeq and A Visualized Phenotype Description Network for AD, BC, and LC. This file includes the embedding visualization of phenotype descriptions utilized in PheSeq. Additionally, it also introduces the web service for visualizing the phenotype description network mentioned in the paper.

## Data Availability

The authors claim that all datasets on which the conclusions of the paper rely are deposited in publicly available repositories. The AD GWAS summary data of IGAP can be downloaded from https://www.niagads.org/system/tdf/public_docs/IGAP_summary_statistics.zip?file=1 [103]. The link for BC transcriptome data is https://tcga-xena-hub.s3.us-east-1.amazonaws.com/download/TCGA.BRCA.sampleMap/AgilentG4502A_07_3.gz [104]. The link for LC prognostic data is https://tcga-xena-hub.s3.us-east-1.amazonaws.com/download/TCGA.LUNG.sampleMap/HumanMethylation450.gz [104]. The three processed sequence analysis data can be downloaded from https://github.com/bionlp-hzau/PheSeq/tree/main/HeterogeneousData/P-ValeData [105]. For the edge files of gene-diseases association networks mentioned in this work, STRING-PPI can be downloaded at https://github.com/xiangyue9607/BioNEV/tree/master/data/STRING_PPI [43]. The PPI network node2vec_PPI can be downloaded at https://github.com/xiangyue9607/BioNEV/tree/master/data/node2vec_PPI [48]. In addition, Mashup_PPI, is downloadable by https://github.com/xiangyue9607/BioNEV/tree/master/data/Mashup_PPI [49]. The link to BioPlax 3.0 is https://bioplex.hms.harvard.edu/interactions.php [51], HuRI can be downloaded from http://www.interactome-atlas.org/download [52], and the drug-target networks can be downloaded from https://github.com/bionlp-hzau/PheSeq/blob/main/HeterogeneousData/EmbeddingData/GraphData/SupplementaryGraphEmbedding/edge_supp_dir/41587_2007_BFnbt1338_MOESM6_ESM.xls [106]. The pre-computed embedding files for all six networks can be downloaded from https://github.com/bionlp-hzau/PheSeq/tree/main/HeterogeneousData/EmbeddingData/GraphData/SupplementaryGraphEmbedding [105]. The literature annotation files containing gene and phenotype descriptions are available for downloading at https://drive.google.com/file/d/1EjqsiFvT4acuSmv FkfWuSRZ2DIfdYvjJ/view?usp=sharing [107]. The text annotation files and pre-computed embedding files of 32 pan-cancers can be downloaded from http://lit-evi.hzau.edu.cn/PheSeq/more-diseases [108]. The source codes and example data are publicly available at https://github.com/bionlp-hzau/PheSeq/tree/main [105]. A user-friendly web page (http://lit-evi.hzau.edu.cn/PheSeq [108]) provides the visualized phenotype description network and all the phenotype description and association significance data in three case studies. In addition, pre-computed embedding representation and association description for 32 specific cancer types is also provided. For a wide range of users who are aiming to investigate an aimed disease by using PheSeq, data observation is suggested by the proposed visualization methods. The data formatting, code pipeline, and result analysis are suggested in Additional file 3. Additional file 2: Appendix A.0.3.

## Footnotes

1 Webpage of the visualized phenotype description network. http://lit-evi.hzau.edu.cn/PheSeq [108]
