# Supplementary material for: PheSeq, a Bayesian deep learning model to enhance and interpret the gene-disease association studies
Source: Genome Med. 2024 Apr 16;16:56. doi: 10.1186/s13073-024-01330-7 (PMC11020195; doi:10.1186/s13073-024-01330-7)
Supplement: Supplementary file 1 — Additional file 1. Usage Guideline of PheSeq Code. This file provides detailed parameter descriptions and command line usage instructions for all scripts involved in the PheSeq. [file 13073_2024_1330_MOESM1_ESM.pdf]

Additional file 1: Usage Guideline of PheSeq Code

Codes Availability

All code configuration and usage details of the PheSeq model can be found on GitHub `https://github.com/bionlp-hzau/PheSeq`.

PheSeq Installation, Environment Setting, and the Whole Pipeline

The folder of src_DynamicMetaEmbeddings contains the model code for PheSeq model training using the dynamic meta-embedding methods.

PheSeq is coded using Python 3.7 on Ubuntu 21.04. The server utilized in our experiments comprises an 11th Gen Intel(R) Core(TM) i7-11700K processor with 16 cores and two NVIDIA GeForce 3090 GPUs. The PheSeq implementation relies on several python packages, i.e., torch v.1.7.1, sympy v. 1.8.0, scipy v. 1.1.2, Transformers v. 4.10.2, NumPy v. 1.19.5, spaCy v. 2.3.5, and sci-kit-learn v. 0.20.0.

Other dependency packages can be found in the requirement.txt, and batch installed by the command line with

```
pip3 install -r requirements.txt.
```

All the provided codes for PheSeq consists of two pipelines, i.e., phenotypic embedding generation pipeline, and PheSeq data fusion pipeline (**Additional file 1: Fig.** S1).

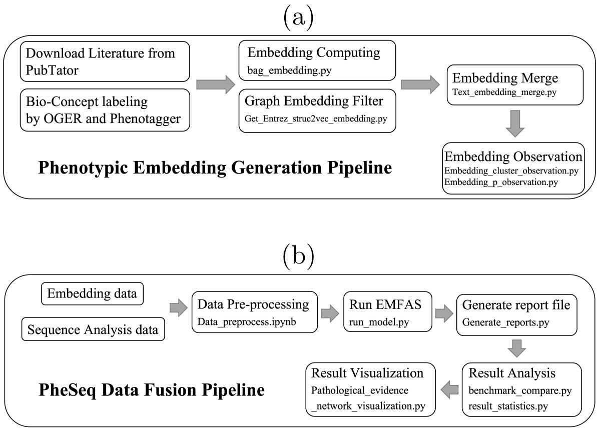

Figure S1: **Code pipeline of the PheSeq model.** **(a)** Codes pipeline for embedding data generation; **(b)** Codes pipeline for candidate gene prioritization.

Phenotypic Embedding Generation Pipeline

It is highly recommended to follow the data construction methods used to retrieve the traceable textual evidence and biomedical concept associations from PheSeq.

For the embedding data construction. The construction methods of embedding data are recorded in HeterogeneousData/EmbeddingData/README.md. The users can follow the embedding construction methods used in this research according to **Additional file 1: Fig.** S1(a).

First, for collecting all literature data related to a disease, PubTator API makes the data query and downloading smoothly (`https://www.ncbi.nlm.nih.gov/research/pubtator/`) . Second, for all biomedical entities mentioned in the literature, the BERT-based NER model trained in the AGAC corpus is used for biomedical events, such as molecular physiological activity, cell physiological activity, and interaction. The codes and guidelines are available at `https://github.com/bionlp-hzau/BERT-for-BioNLP-OST2019-AGAC-Task1`. Additionally, OGER++ is used to annotate the GO terms, and OGER++ can be found at `https://github.com/OntoGene/OGER`. While PhenoTagger is used to annotate the HPO terms, the source code can be found at `https://github.com/ncbi-nlp/PhenoTagger`. In addition, PubTator is used to annotate the Gene, disease, proteins, and the API documents can be found in `https://www.ncbi.nlm.nih.gov/research/pubtator/api.html`.

This step can be adjusted if you have any more annotation familiar tools or other biomedical entities of interest. Note that existing biomedical entities annotated by PubTator will also be retained.

For graph embedding, the relevant data are downloaded from `http://web.cse.ohio-state.edu/yue.149/BioNEV` The graph embedding data and node data downloaded will be pre-processed in the subsequent step.

After this, bag_embedding.py is used to construct embeddings for text and biomedical concepts respectively using the pre-trained bio-BERT model. This code runs with the following command line and options:

bag_embedding.py runs with the following operation:

```
python bag_embedding.py --input [INPUT_FILE]
--output [SAVE_FILE]
--max_len [MAX_LEN]
--max_bag_size [MAX_BAG_SIZE]
--embedding_size [EMBEDDING_SIZA]
--model [MODEL]
--use_cpu
--single_sentence_file [SINGLE_SENTENCE_FILE]
--text_norm
```

bag_embedding.py runs with the following options:

```
[INPUT_FILE]: the literature file or biomedical
    concept file for embedding calculation. The
    example files can be found in
    HeterogeneousData/EmbeddingData/TextData.
[SAVE_FILE]: Precomputed embedding file for
    model input.
[MAX_LEN]: The length limitation of the input
    sentences.
[MAX_BAG_SIZE]: The limitation of the sentence
    number for each association used for
    embedding computation.
```

```
[EMBEDDING_SIZA]: The dimension of the
    precomputed embedding for each association.
[MODEL]: The model version of BERT used for
    embedding computation. "base" for
    "dmis-lab/biobert-base-cased-v1.1" and
    "large" for
    "dmis-lab/biobert-large-cased-v1.1".
[use_cpu]: Using CPU to compute the embedding or
    not (GPU).
[SINGLE_SENTENCE_FILE]: The input file for
    embedding computation is the "bio-concept"
    file or the "literature file" defined in
    GitHub.
[text_Norm]: Using text normalization or not.
```

And Get_Entrez_struc2vec_embedding.py is used to filter the graph embedding data according to the provided Entrez list. This code runs with the following command line and options. Get_ Entrez_ struc2vec_ embedding.py runs with the following operation:

```
python Get_Entrez_struc2vec_embedding.py
--gene2ensemble_file [gene2ensemble_file]
--node_file [node_file]
--embedding_file [embedding_file]
--save_file [save_file]
```

Get_Entrez_struc2vec_embedding.py runs with the following options:

```
[gene2ensemble_file]: The gene-ensemble ID
    mapping file.
[node_file]: The nodes file of the graph
    embedding.
[embedding_file]: The graph embedding file.
[save_file]: The file used to save the processed
    graph embedding.
```

We also provide the script to calculate the gene embeddings from different networks, node2vec_embedding_generation.py. This script allows for use in providing edge files to quickly construct network embeddings of genes using the node2vec algorithm. Note that gene identifiers need to be normalized to entrez IDs to facilitate subsequent processing.

node2vec_embedding_generation.py runs with the following operation:

```
python node2vec_embedding_generation.py
--edge_file [edge_file]
--save_file [save_file]
--embedding_size [embedding_size]
```

node2vec_ embedding_ generation.py runs with the following options:

```
[edge_file]: Tab-splited edge file, each line
    includes two nodes.
[save_file]: File path used to save the
    calculated network embeddings.
[embedding_size]: The size of calculated
    embeddings.\\
```

Finally, Gene_embedding_merge.py is used to merge the text embeddings, biomedical entity embeddings, and graph embeddings, to obtain the complete embedding data with rich semantic information. This code runs with the following command line and options:

Gene_embedding_merge.py runs with the following operation:

```
python Gene_embedding_merge.py
--text_embedding_file [text_embedding_file]
--concept_embedding_file [concept_embedding_file]
--graph_embedding_file [graph_embedding_file]
--embedding_save_file [embedding_save_file]
```

Gene_embedding_merge.py runs with the following options:

```
[text_embedding_file]: The text embedding file
    generated from "Text Embedding Calculation".
[concept_embedding_file]: The concept embedding
    file generated from "Text Embedding
    Calculation".
[graph_embedding_file]: The graph embedding file
    of genes, the alternative gene network
    embeddings can be replaced here.
[embedding_save_file]: The averaging-summed
    embedding file.\\
```

There are alternative gene network embeddings provided in the repository, including node2vec_PPI, Mashup_PPI, BioPlex 3.0, HuRI, and Drug-Target. More information on these network embeddings can be found in the GitHub repository. Users are also allowed to use their own pre-trained embeddings for replacement.

The p-value data construction. The construction methods of p-value data can be found in HeterogeneousData/ P-ValueData/ README.md.

The GWAS Summary data for AD are collected from GWAS Catalog (`https://www.ebi.ac.uk/gwas/`), and both transcriptome data for BC and methylation data for LC are collected from TCGA (`https://www.cancer.gov/about-nci/organization/ccg/research/structural-genomics/tcga`).

After collecting gene-p-value data, the NCBI gene-Entrez mapping file (`https://ftp.ncbi.nih.gov/gene/DATA/gene_info.gz`) needs be used for converting gene symbol to Entrez ID.The processed p-value data needs to have three columns separated by Tab keys, including GeneSymbol, EntrezID and p-value, the file format is the same as the "P-ValueData/ BC/BC.symbol_entrez.p.tsv".

If p-value data for other diseases need to be collected, the same collection methods can be taken, i.e. GWAS data in the GWAS Catalog and other sequence analysis data in the TCGA database. Note that p-value data need to be processed as tab-delimited three-column files containing gene symbol, Entrez ID, and p-value respectively.

Data pre-processing. When the text embedding, biomedical entity embedding, and graph embedding are constructed, the Text_embedding_merge.py is used

to merge the three types of embeddings to obtain the final embedding data.

By running this code, two pre-processed data files can be obtained.

Summary data includes the sentence descriptions and p-value for each gene, and example files can be found in HeterogeneousData/EmbeddingData/TextData. The Summary file format is as follows (Tab separated):

```
GENE_LINE: $GENE_Symbol $Entrez_ID $p-value
$PMID_1 Sentence_1 {$Tag_1, Tag_2}
$PMID_2 Sentence_2 {$Tag_1, Tag_2}
```

Embedding data can be derived from different representation learning methods, such as Graph embedding and text embedding. Embedding files format is as follows (Tab-separated):

```
$Entrez_ID_1 $embedding_vec_1
$Entrez_ID_2 $embedding_vec_2
```

Embedding Data Observation. To facilitate the observation of congruence between p-values and embedded representation data, we provide a visualization tool to visualize the associations. The embedding observation code as shown in **Additional file 1: Fig.** S1 and **Additional file 1: Fig.** ?? is available in GitHub, and the command line is as follows.
Embedding_cluster_observation.py is used to display the embedding clustering results and requires two files.

```
python Embedding_cluster_observation.py -cf
    [cluster_file]
-ef [embedding_file]
-sp [save_path]
```

Embedding_cluster_observation.py runs with the following options:

```
[cluster_file]: a file containing the embedding
    name and the corresponding cluster name,
    tab-separated.
[embedding_file]: a file containing the
    embedding name and the corresponding
    embedding vector, tab-separated.
[save_path]: path for saving figure.
```

Embedding_p_observation.py is used to observe the intrinsic association mechanism between the embedded data and the p-values.

```
python Embedding_p_observation.py -pf
    [p_value_file] -ef [embedding_file] -sp
    [save_path]
```

Embedding_cluster_observation.py runs with the following options:

```
[p_value_file]: A file contains the embedding
    name and the p-value, tab key delimited.
[embedding_file]: a file containing the
    embedding name and the corresponding
    embedding vector, tab-separated.
[save_path]: path for saving figure.
```

We recommend users to employ Static-PheSeq when the embeddings are observed to exhibit high quality. Nevertheless, in cases where the embeddings demonstrate, Dynamic-PheSeq may yield superior results.

In addition, for users running PheSeq with their own p-value data and phenotype description data, it is suggested to observe the congruence of the two types of data with this observation method. The global view formed from the figure also helps to decide a proper threshold for the p-value in terms of data congruence.

PheSeq Data Fusion Pipeline

After data pre-processing, the Static-PheSeq and Dynamic-PheSeq models for the gene-disease association inference pipeline can be run according to **Additional file 1: Fig.** S1.

Command line and option setting for Static-PheSeq.

```
python src/run_model.py -ef [embedding_file]
-sf [summary_file]
-lp [log_save_path]
-lf [log_prefix]
-mh [multi_hidden]
--rs [random_seed]
-ed [embedding_size]
-lr [learning_rate]
-hd [hidden_dim]
-tt [train_time]
-bs [batch_size]
-pt [p_value_threshold]
```

Static-PheSeq runs with the following options:

```
[embedding_file]: The precomputed embedding file.
[summary_file]: The summary file, file format is
    described in the GitHub project.
[log_save_path]: The folder used to save the log
    file.
[log_prefix]: The name prefix for the saved log
    file.
[multi_hidden]: Use the multi-hidden layers
    module in the deep learning part.
[random_seed]: Random seed.
[embedding_size]: The dimension of the input
    embedding.
[learning_rate]: The learning rate.
    [hidden_dim]: 50, the dimension of hidden
    layer.
[train_time]: Training epoch.
[batch_size]: Batch size.
[p_value_threshold]: The significance threshold
    of the p-value.
```

Command line and option setting for Dynamic-PheSeq.

```
python src/Dynamic_PheSeq.py -mf [summary_file]
    -ef [embedding_file]
-ds [data_size]
-ed [embedding_size]
```

```
-mh [multi_hidden]
-hs [hidden_dim]
-zd [z_dim]
-bs [batch_size] -tt [train_time]
-lr [learning_rate]
-mr [mu_learning_rate]
-sr [sigma_learning_rate]
-rs [random_seed]
-ga [gradient_accumulation]
-sl [save_log]
-lp [log_save_path]
-lf [log_save_prefix]
-uf [use_fake_data]
```

Dynamic-PheSeq runs with the following options:

```
[summary_file]: The summary file, file format is
    described in "HeterogeneousData
    /EmbeddingData /README.md".
[embedding_file]: The embedding file.
[data_size]: The data size (genotype-phenotype
    association count) of the input data.
[embedding_size]: The embedding dimension of the
    embedding data.
[multi_hidden]: Use more hidden layers.
[hidden_dim]: The hidden layer dimension of the
    deep learning module.
[z_dim]: The dimension of the latent variable
    $Z$.
[log_save_path]: The folder used to save the log
    file.
[log_prefix]: The name prefix of the log file.
[batch_size]: Data count of each mini-batch.
[train_time]: The training epoch.
[batch_size]: Batch size.
[learning_rate]: The learning rate.
[mu_learning_rate]: The learning rate for
    $\mu$-related part in the deep learning
    module.
[sigma_learning_rate]: The learning rate for
    $\sigma$-related part in the deep learning
    module.
[random_seed]: Random size.
[gradient_accumulation]: Using gradient
    accumulation strategy in model training or
    not.
[save_log]: Save the log file or not.
[log_save_path]: The folder for saving the log
    file.
[log_save_prefix]: The name prefix for the saved
    log file.
[use_fake_data]: True, using test file or not.
```

PheSeq prioritization results analysis. Referring to **Additional file 1: Fig.** S1, after prioritizing the disease candidate genes by PheSeq, the results can be further analyzed and visualized by the following code.

The Generate_report.py is used to generate report file. This code runs with the following command line and options:

Generate_report.py runs by the following operation:

```
python Generate_report.py --log_path [log_path]
--summary_path [summary_path]
--output_path [output_path]
--output_prefix [output_prefix]
--diseases_source [diseases_source]
```

Generate_report.py runs by the following options:

```
[log_path]: The folder includes the PheSeq
    prediction result.
[summary_path]: The folder includes a summary
    file generated from Data_preprocess.ipynb.
[output_path]: The folder used to save the
    result.
[output_prefix]: The name prefix for the result
    file.
```

The benchmark_compare.ipynb is a Jupyter notebook code that compares the PheSeq prediction results with the existing knowledge of DISEASES and plots simple cumulative curves and calculations of evaluation metrics such as MRR.

Finally, the Gene-Evidence-GO-HPO_Statistics.ipynb is also a Jupyter notebook code that counts the number of traceable textual evidence, counts the number of various biological concepts, and draws a bar chart for data statistics.

Obtain a visualized phenotype description network. To facilitate the mechanism investigation of the prioritized genes by PheSeq, a script is provided to automatically generate the visualized phenotype description network web page. This script allows the input of a report file, generated by PheSeq, and a list of significant genes obtained by sequence analysis. By automatically counting the number of associations between these genes and HPO/GO concepts, web page files are created via Echarts.
The phenotype description network is visualized by the following command line:

```
python src_for_result/
    Pathological_evidence_network_visualization.py
    --report_file [report_file]
-evidence_file [evidence_file]
--save_file [save_file]
--topn [topn]
--tax_id [tax_id]
--sequence_analysis_gene_file
    [sequence_analysis_gene_file]
--filter_keyword [filter_keyword]
```

Pathological_evidence_network_visualization.py runs by the following options:

```
[report_file]: The folder used to save the
    PheSeq prediction result.
[evidence_file]: The folder includes the summary
    file generated from Data_preprocess.ipynb.
[save_file]: The file used to save the result.
[topn]: Top-n association for the report.
[tax_id]: The tax id for the target species.
[sequence_analysis_gene_file]: File path for the
    summary file.
[filter_keyword]: A keyword used to filter the
    reported result .
```
