# Supplementary material for: PheSeq, a Bayesian deep learning model to enhance and interpret the gene-disease association studies
Source: Genome Med. 2024 Apr 16;16:56. doi: 10.1186/s13073-024-01330-7 (PMC11020195; doi:10.1186/s13073-024-01330-7)
Supplement: Supplementary file 2 — Additional file 2. Model Solution and Implementation. This file contains comprehensive algorithmic solutions implemented in PheSeq, detailing the models and their implementations. [file 13073_2024_1330_MOESM2_ESM.pdf]

Additional file 2: Model Solution and Implementation

Tutorial Video of the PheSeq Model

The tutorial video is released in Youtube, `https://youtu.be/GXlej3WAoM4`.

Model Framework

Under a Bayesian generative model framework, we proposed PheSeq. PheSeq regards association significance with its uncertainty through a random variable setting in a Bayesian network, integrates the phenotype description in the form of the phenotypic embedding, feeds the heterogeneous data into a Bayesian framework, and pinpoints vital gene-disease associations after data fusion.

According to the input type, the model has two forms, i.e., a Static-PheSeq for a set of fixed embedding data, and a Dynamic-PheSeq for a set of flexible and learnable embedding data.

The Static-PheSeq model assumes that the embedding data are already well-learned to represent the source data, so a fixed deep-learned Representation is fed into the Bayesian graphical model and captures the potential relations and dependencies among the genotype-phenotype data. It is straightforward to obtain the parameter iterations via the standard maximum likelihood estimate (MLE) computation, and obtain the optimal estimation of graphical model parameters;

Unlike the Static-PheSeq model which takes fixed embedding data, the Dynamic-PheSeq model involves both phenotypic embedding and p-value into a BDL framework. The Static-PheSeq includes a deep learning module to capture high-quality representations and a graphical model module to learn the potential relation dependencies among observations. This model is solved by a maximum-a-posterior (MAP) estimation strategy, and embedding data and all the parameters are dynamically adjusted.

Static-PheSeq Model and Its Solution via MLE

Model and parameter setting

The observation data, latent variables, and model parameters in this PheSeq model are displayed in **Additional file 2: Fig.** S1, where the shaded circles represent observations, while the blue circles represent latent variables and model parameters. All the variables used in this model are denoted as below:

$$\begin{cases} \text{Obs: } Z = \{Z_g\}, P = \{P_g\}, g = 1, \cdots, G, \\ \text{Latent variables: } F = \{F_g\}, T = \{T_g\}, g = 1, \cdots, G, \\ \text{Model parameters: } \Theta = \{\phi, \alpha_g, a_g, b_g\}, g = 1, \cdots, G. \end{cases}$$

For a given disease d and each gene g, the observation data include association significance data P_g and phenotypic embedding data Z_g. The former are collected from sequence analysis and the latter is learned from a neural network $\mathcal{V}_\theta(\cdot)$ by using the association descriptions L_g, which are collected from text/graph.

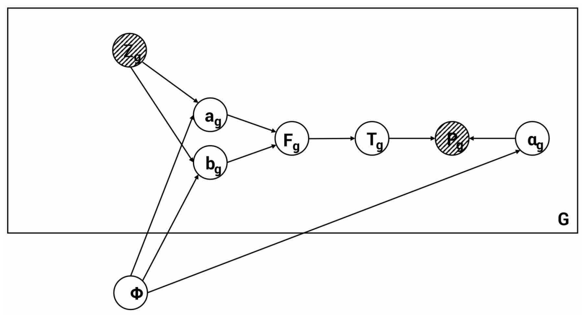

Figure S1: **Graph of conditional dependency structure for observations and latent variables in Static-PheSeq model.** The shaded circles refer to all the observation data, including Lg and Pg for a given gene g, and other circles represent the other variables, including the latent variables a_g, b_g, F_g, T_g and α_g, and the neural network parameters ϕ.

There are two latent variables imported in Static-PheSeq, i.e., T_g, P_g, and F_g, the distributions of which are defined below:

$$\begin{cases} F_g \sim Beta(a_g, b_g), \\ T_g \sim Bernoulli(F_g), \\ P_g \sim T_g Beta(\alpha_g, 1) + (1 - T_g) U(0, 1), \end{cases} \quad (1)$$

Here, $F = \{F_g\}$ is a latent variable that represents the scores of gene-disease association and follows the beta distribution with a_g and b_g as parameters, $F_g \sim \text{Beta}(a_g, b_g)$.

In addition, T_g is another latent variable that plays a switch role by taking a binary value, where 1 indicates a *g-d* association and 0 indicates a non-association. Using the switch property of T_g, the distribution of P_g is modeled by pdf in (1). When the switch is on (T_g=1), P_g follows a beta distribution, Beta(α_g, 1). Here, α_g is generated by ϕ and prone to be close to zero, which leads to a significant association between gene g and disease d. Conversely, when the switch is off (T_g=0), P_g follows a uniform distribution of U(0, 1) and makes it a high chance to sample a less significant p-value.

Finally, all the parameters are denoted as a parameter set Θ. Θ includes $\alpha_{g(1:G)}, a_{g(1:G)}$, and $b_{g(1:G)}$, which are distribution parameters as defined in (1). In addition, Θ includes ϕ, which denotes the parameters of the neural network f, into which the embedding data Z_g is fed. Therefore, the model parameters a_g and b_g are computed through Z_g and f, i.e., $(a_g, b_g) = f_\phi(Z_g)$.

Joint probability $p(P, T, F|Z, \Theta)$ *and variational distribution function for latent variables* $q(T, F)$

Owing to the dependence among observation and latent variables as shown in **Additional file 2: Fig.** S1,

the joint probability density function, $p(P,T,F|Z,\Theta)$, is decomposed as:

$$p(P,T,F|Z,\Theta) = p(P|T,\Theta)p(T|F,\Theta)p(F|Z,\Theta), \quad (2)$$

where $\Theta = \{\phi, \alpha(\phi), a(\phi), b(\phi)\}$. Through straightforward calculation, we have $p(P_g|T,\Theta) = T\alpha P_g^{\alpha-1} + (1-T)$.

If $T_g = 1$, $p(P_g|T,\Theta) = \alpha P_g^{\alpha-1} = (\alpha P_g^{\alpha-1})^{T_g}$. Conversely, $T_g = 0$, $p(P_g|T,\Theta) = 1$. Therefore, we obtain

$$p(P_g|T,\Theta) = (\alpha P_g^{\alpha-1})^{T_g}. \quad (3)$$

From the distribution of T in (1), we have

$$p(T_g|F,\Theta) = F_g^{T_g}(1-F_g)^{1-T_g}. \quad (4)$$

Similarly, the distribution of F in (1) yields to

$$p(F_g|Z,\Theta) = \frac{\Gamma(a_g+b_g)}{\Gamma(a_g)\Gamma(b_g)} F_g^{a_g-1}(1-F_g)^{b_g-1}. \quad (5)$$

Substituting (3), (4), and (5) into (2), we have

$$\begin{aligned} p(P,T,F|Z,\Theta) &= p(P|T,\Theta)p(T|F,\Theta)p(F|Z,\Theta) \\ &= \prod_{i=1}^{G}[(\alpha_i P_i^{\alpha_i-1})^{T_i} F_i^{T_i}(1-F_i)^{1-T_i} \frac{\Gamma(a_i+b_i)}{\Gamma(a_i)\Gamma(b_i)} \\ &\quad F_i^{a_i-1}(1-F_i)^{b_i-1}]. \end{aligned} \quad (6)$$

From the standard mean-field variational inference method, we assume that the latent variables, T, F, follow a variational distribution under pdf $q(T,F)$. The decomposition rule yields to $q(T,F) = \prod_{g=1}^{G} q_g(T_g,F_g)$. In addition, the computation trick in the mean-field variational inference method also derives the optimization of $q_g(T_g,F_g)$ as follows:

$$\begin{aligned} \log q_g^*(T_g,F_g) &= E_{\prod_{i\neq g}^{G} q_i(T_i,F_i)}[\log p(P,T,F \mid Z,\Theta)] \\ &= \sum_i^G E_{\prod_{i\neq g}^{G} q_i(T_i,F_i)}[T_i \log\alpha_i + T_i(\alpha_i-1)\log P_i \\ &\quad +(T_i+\alpha_i-1)\log F_i + \log\frac{\Gamma(a_i+b_i)}{\Gamma(a_i)\Gamma(b_i)} \\ &\quad +(b_i-T_i)\log(1-F_i)] \\ &= T_g\log\alpha_g + T_g(\alpha_g-1)\log P_g + (T_g+a_g-1)\log F_g \\ &\quad +(b_g-T_g)\log(1-F_g) + C. \end{aligned}$$

where $C = -\log((1-F_g) + F_g\alpha_g P_g^{\alpha_g})$.

Henceforth, we have

$$q_g^*(T_g,F_g) = \alpha_g^{T_g} P_g^{T_g(\alpha_g-1)} F_g^{(T_g+\alpha_g-1)}(1-F_g)^{(b_g-T_g)} C'.$$

while $C' = \exp(C)$. [1]

[1] One can directly show that $q_g^*(T_g,F_g)$ is a pdf: $\int\sum_{T_i=0}^{1}(\alpha_i P_i^{\alpha_i-1})^{T_i} F_i^{T_i}(1-F_i)^{1-T_i} dP_i = \int 1 - F_i + F_i\alpha_i P_i^{\alpha_i-1} dP_i = (1-F_g)P_g\Big|_0^1 + (F_g\alpha_g P_g^{\alpha_g-1})P_g\Big|_0^1 = 1 - F_g + F_g = 1$.

Loss function and gradient computation with respect to ϕ

From (6), the logarithm of the joint probability function is

$$\begin{aligned} \log p(P,T,F|Z,\Theta) &= \log\prod_{i=1}^{G} p(P_i,T_i,F_i|Z_i,\Theta) \\ = \sum_{i=1}^{G} &[T_i\log\alpha_i + T_i(\alpha_i-1)\log P_i + (T_i+a_i-1)\log F_i \\ &+ (b_i-T_i)\log\Big(1-F_i\Big) + \log\frac{\Gamma(a_i+b_i)}{\Gamma(a_i)\Gamma(bi)}\Big]. \end{aligned}$$

For the MLE algorithm, the goal is to maximize the likelihood function. Taking the variational sampling of the latent variable into consideration, a loss function is defined as the expectation of the logarithm of the joint pdf

$$L(\phi) = E_{q(T,F)}[\log p(P,T,F \mid Z,\Theta)]. \quad (7)$$

A Monte-Carlo estimation of (7) leads to

$$\widetilde{L}(\phi) = \log p(P,T^*,F^* \mid Z,\Theta), \text{ where } T^*,F^* \sim q^*(T,F).$$

As $\nabla_\phi L(\phi) \approx \nabla_\phi\widetilde{L}(\phi)$, we have the noisy estimate of the gradient with respect to the neural network parameters, ϕ.

$$\nabla_\phi\widetilde{L}(\phi) = \sum_{g=1}^{G}(\nabla_{\alpha_g}\frac{\partial\alpha_g}{\partial\phi} + \nabla_{a_g}\frac{\partial a_g}{\partial\phi} + \nabla_{b_g}\frac{\partial b_g}{\partial\phi}),$$

where

$$\begin{cases} \nabla_{\alpha_g} = T_g^*(\log P_g + \frac{1}{\alpha_g}), \\ \nabla_{a_g} = \frac{\Gamma(a_g+b_g)\Psi(a_g+b_g)-\Gamma(a_g+b_g)\Psi(a_g)}{\Gamma(a_g+b_g)} + \log F_g^*, \\ \nabla_{b_g} = \frac{\Gamma(a_g+b_g)\Psi(a_g+b_g)-\Gamma(a_g+b_g)\Psi(b_g)}{\Gamma(a_g+b_g)} + \log(1-F_g^*). \end{cases}$$

Here, $\Psi(x) = \frac{\Gamma(x)'}{\Gamma(x)}$ is the Digamma function. Eventually, a gradient ascent iteration, $\phi^{(t+1)} = \phi^{(t)} + \eta\nabla_\phi\widetilde{L}(\phi)$, is applied, where η is the learning rate.

Pseudocode of the solution to the Static-PheSeq model

We now propose the solution of the model in the form of the pseudo code in Algorithm 1:

Dynamic-PheSeq Model and Its Solution via MAP-MLE

In Dynamic-PheSeq, the BDL framework is introduced to help the model capture the hidden relationships between the data with dynamic optimized data representation, meanwhile, the graphical model and deep network model parameters are iterations simultaneously.

Algorithm 1 MLE for Static-PheSeq

Require: Annotated text of G genes: Z_g, GWAS p-value of G genes: P_g, Time step: Time
Ensure: optimal ϕ^*, α_g^*

```
Initiate α^(0), φ^(0);
Run neural network f_φ (Z_g)
for t = 0 to Time do
    for each gene g do
        a_g, b_g = f_{φ^t} (Z_g);
        F_g ~ Beta (a_g, b_g)
        T_g ~ Bernoulli (F_g)
        P_g ~ T_g Beta (α^(t), 1) + (1 − T_g) U (0, 1)
        ∇_α calculation
        α^(t+1) = α^(t) − η∇_α
        ∇_{a_g}, ∇_{b_g}, ∂a_g/∂φ^(t), ∂b_g/∂φ^(t) calculation and gradient accumulation
    end for
    φ^(t+1) = φ^(t) − η∇_φ
end for
```

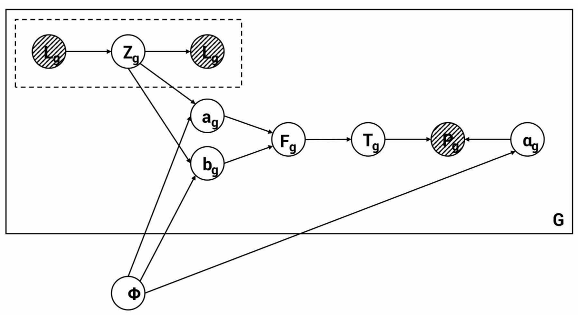

Figure S2: **Graph of conditional dependency structure for observations and latent variables in Dynamic-PheSeq model.** The shaded circles refer to all the observation data, including Lg and Pg for a given gene g, and other circles represent the other variables, including the latent variables Z_g, a_g, b_g, F_g, T_g and α_g, and the neural network parameters ϕ. The VAE module is introduced to dynamically optimize the data representation and the deep learning module and graphical model parameters are iterated simultaneously.

Graphical model and parameter setting

The parameter setting of the graphical model module in Dynamic-PheSeq is consistent with that in Static-PheSeq, and the graphical model is shown in **Additional file 2: Fig.** S2. Compared with Static-PheSeq, the only difference in this graph is the importing of the learnable embedding data Z_g, which is encoded by the input data L_g in a variational autoencoder (VAE) framework. Therefore, the observations, latent variables, and model parameters are denoted in the following.

$$\begin{cases} \text{Obs: } L=\{L_g\}, P=\{P_g\}, g=1,\cdots,G, \\ \text{Latent variables: } Z=\{Z_g\}, F=\{F_g\}, T=\{T_g\}, \\ \text{Model parameters: } \Theta=\{\phi,\alpha_g(\phi),a_g(\phi,Z),b_g(\phi,Z)\} \\ \quad g=1,\cdots,G. \end{cases}$$

In Dynamic-PheSeq, the distributions of latent variables are the same as those in Static-PheSeq, as defined in (1). It should be noted that the model parameters, a and b, are relevant to the input learned embedding Z and the neural network with parameter ϕ, thus denoting it as $a(\phi, Z)$ and $b(\phi, Z)$, respectively. In addition to replacing the fixed embedding input L_g with a learnable Z_g, the rest of the model setting is almost unchanged, if compared with that in Static-PheSeq.

Deep learning setting for decoding learnable embeddings

The dynamic property of Dynamic-PheSeq comes from the flexible encoding of a learnable embedding Z_g. As shown in **Additional file 2: Fig.** S2, a generative latent variable model, VAE, is introduced in the dynamic PheSeq model, which is used to learn a $K-$tuple latent variable Z_g for each gene g, and Z_g has a prior distribution: $p(Z_g) = \mathcal{N}(0, I_K)$.

Under the reparameterization strategy of VAE, $Z_g = \mu_g + \sigma_g \cdot \epsilon_g$ and $\epsilon_g \sim \mathcal{N}(0, I_K)$, the variational distribution with shared network parameters θ of each z_g is given by $q(Z_g|\theta_z) = \mathcal{N}(\mu_\theta(L_g), \sigma_\theta^2(L_g))$.

Since the phenotypic embedding L_g is reconstructed from its latent variable Z_g through the decoding part of VAE which is parameterized by θ, the generation distribution is modeled via a θ, Z-related PDF, denoted by $L_g \sim p_\theta(L_g|Z_g)$.

In the BDL scenario, L_g is a real-valued embedding vector and its prior distribution comes from a Normal distribution $\mathcal{N}(\mathcal{V}_\theta(Z), \lambda^{-1}I)$, where $\mathcal{V}_\theta(Z)$ denotes the decoding part of the VAE network parameterized by θ.

Under the encode/decode mechanism, the learnable embedding Z is first derived from an inference process in the VAE encoder, and it is subsequently fed into a generation process in the VAE decoder to reconstruct L. First, the inference process is implemented as below.

Step-1 For each layer l of the VAE encoder:

1). For each column n of the weight matrix W_l, draw

$$W_{l,*n} \sim \mathcal{N}(0, \lambda_w^{-1} I_{K_l}).$$

2). Draw the bias vector $b_l \sim \mathcal{N}(0, \lambda_w^{-1} I_{K_l})$.

3). For each row g of h_l, draw

$$h_{l,g*} \sim \mathcal{N}(\sigma(h_{l-1,g*} W_l + b_l), \lambda_s^{-1} I_K).$$

Step-2 For each gene g:

1). Draw latent mean and covariance vector

$$\mu_g \sim \mathcal{N}(h_L W_\mu + b_\mu, \lambda_s^{-1} I_K),$$
$$\log \sigma_g^2 \sim \mathcal{N}(h_L W_\sigma + b_\sigma, \lambda_s^{-1} I_K).$$

2). Draw latent vector

$$Z_g \sim \mathcal{N}(\mu_g, diag(\sigma_g)).$$

In the above, $\lambda_w, \lambda_s, \lambda_x$ are hyperparameters in VAE. Furthermore, the generation process of L_g

through the decoder of VAE is implemented as follows:

Step-1 For each layer l of the encoder

1). For each column n of the weight matrix W_l, draw

$$W_{l,*n} \sim \mathcal{N}(0, \lambda_w^{-1} I_{K_l}).$$

2). Draw the bias vector $b_l \sim \mathcal{N}(0, \lambda_w^{-1} I_{K_l})$.

3). For each row g of h_l, draw

$$h_{l,g*} \sim \mathcal{N}(\sigma(h_{l-1,g*} W_l + b_l), \lambda_s^{-1} I_K).$$

Step-2 For reconstructed L_g, draw

$$L_g \sim \mathcal{N}(h_L W_{L+1} + b_{L+1}, \lambda_x^{-1} I). \tag{8}$$

A.0.1 Joint probability $p(P, L, F, T, Z|\Theta)$ and variational distribution function for latent variables $q(F, T, Z)$

Owing to the dependence among observation and latent variables as shown in **Additional file 2: Fig.** S2, the joint probability density function, $p(P, L, F, T, Z|\Theta)$, is decomposed as:

$$\begin{aligned} &p(P, T, F, Z, L|\Theta) = \\ &\quad p(P|T, \Theta) p(T|F, \Theta) p(F|Z, \Theta) p_\theta(L|Z) p(Z). \end{aligned} \tag{9}$$

The Θ in the formula is the set of all parameters, $\Theta = \{\phi, \alpha(\phi), a(\phi, Z), b(\phi, Z)\}$.

To calculate $p(P_g \mid T, \Theta)$, we also have $p(P_g|T, \Theta) = T\alpha P_g^{\alpha-1} + (1-T)$. If $T_g = 1$, $p(P_g|T, \Theta) = \alpha P_g^{\alpha-1} = (\alpha P_g^{\alpha-1})^{T_g}$. Conversely, $T_g = 0$, $p(P_g|T, \Theta) = 1$. Therefore, we denote:

$$p(P_g|T, \Theta) = (\alpha P_g^{\alpha-1})^{T_g}. \tag{10}$$

From the distribution of T in (1), we again get

$$p(T_g|F, \Theta) = F_g^{T_g} (1-F_g)^{1-T_g}. \tag{11}$$

From the distribution of F in (1), we obtain

$$p(F_g|Z_g, \Theta) = \frac{\Gamma(a_g + b_g)}{\Gamma(a_g)\Gamma(b_g)} F_g^{a_g-1} (1-F_g)^{b_g-1}. \tag{12}$$

Substituting (10), (11) and (12) into (9), we obtain

$$\begin{aligned} &p(P, T, F, Z, L|\Theta) \\ &= p(P|T, \Theta) p(T|F, \Theta) p(F|L, \Theta). \\ &= \prod_{i=1}^{G} [(\alpha_i P_i^{\alpha_i - 1})^{T_i} F_i^{T_i + a_i - 1} (1-F_i)^{b_i - T_i} \tfrac{\Gamma(a_i+b_i)}{\Gamma(a_i)\Gamma(b_i)} \\ &\quad p_\theta(L_g|Z_g) p(Z_g)]. \end{aligned} \tag{13}$$

Here, the inference of this model is difficult to perform since $p_\theta(L|Z)$ is determined by the neural network module with non-linear units. To address this, the variational inference method is used to approximate the posterior probability distribution through the mean field.

$$q(F, T, Z) = \prod_{g=1}^{G} q(F_g|a_g, b_g) q(T_g|F_g) q(Z_g|\mu_\theta(L_g), \sigma_\theta^2(L_g)).$$

MAP algorithms for VAE module parameter optimization

To optimize parameters in the VAE module, coordinate ascent and the MAP algorithms were used to optimize the parameters of the VAE module with fixed PGM parameters, and the evidence lower bound (ELBO) can be obtained by

$$\begin{aligned} L(q) &= E_q[\log p(P|T) + \log p(T|F) + \log p(F|Z) + \log p_\theta(L|Z)] \\ &\quad - \mathbb{KL}(q_\theta(Z|L) \| P(Z)) - E_q[\log q(F)] - E_q[\log q(T)]. \end{aligned}$$

For a given ELBO, MAP is performed by considering the variational distribution of $q_\theta(Z_g|\theta_z)$ and maximizing the objective w.r.t. $\{F_g\}$ using block coordinate ascent. The objective thus becomes the following.

$$\begin{aligned} &L^{MAP}(F_g, T_g, \Theta, \theta) = T_g(\log \alpha_g F_g + (\alpha_g - 1)\log P_g) \\ &\quad + (1-T_g)\log(1-F_g) + \log \tfrac{\Gamma(a_i+b_i)}{\Gamma(a_i)\Gamma(b_i)} \\ &\quad + (a_g - 1)\log F_g + (b_g - 1)\log(1-F_g) \\ &\quad + E_{q_\theta(Z_g|L_g)}[\log p(L_g|Z_g)] \\ &\quad - \mathbb{KL}(q_\theta(Z_g|L_g) \| p(Z_g))) \\ &\quad - \tfrac{\lambda_w}{2} \textstyle\sum_{l=1}^{L} (\|W_l\|_F^2 + \|b_l\|_2^2), \end{aligned} \tag{14}$$

where $\theta = \{\{w_{\{1...L\}}\}, \{b_{\{1...L\}}\}\}$ is the parameters of VAE network, w_l and b_l is the weight and bias of lth layer.

The block coordinate ascent for $\{F_g\}$ thus becomes $F_g \leftarrow \frac{T_g + a_g - 1}{a_g + b_g - 1}$. In terms of $q_\theta(Z_g|L_g)$, isolating the terms related to Z_g in (14) is

$$\begin{aligned} L(\Theta, \theta; Z_g) &= E_{q_\theta(Z_g|L)}[\log p(F|Z) + \log p_\theta(L_g|Z_g)] \\ &\quad - \mathbb{KL}(q_\theta(Z_g|L_g) \| P(Z_g)). \end{aligned}$$

Monte Carlo estimation is used to simplify the expectation calculation and we have

$$\begin{aligned} \tilde{L}(\Theta, \theta; Z_g) &= \tfrac{1}{N} \textstyle\sum_{n=1}^{N} [\log p_\theta(L_g|Z_g^{(n)}) + \log p(F_g|Z_g^{(n)})] \\ &\quad - \mathbb{KL}(q_\theta(Z_g|L_g) \| P(Z_g)). \end{aligned}$$

where the $N = \{1, 2, ..., N\}$ is the Monte Carlo sampling number, and $Z_g^{(n)} = \mu_g + \sigma_g \cdot \epsilon^{g(n)}$ and $\epsilon^{g(n)} \sim \mathcal{N}(0, I_K)$, where $\cdot^{(n)}$ notifies the n-th sampling.

Given F_g, the gradient with respect to μ_g and σ_g of Z_g is

$$\begin{cases} \nabla_{\mu_g} \tilde{L}(\Theta, \theta; Z_g) = \frac{1}{N} \sum_{n=1}^{N} [\nabla_{\mu_g} \log p_\theta(L_g|Z_g^{(n)}) \\ \qquad + \nabla_{\mu_g} \log p(F_g|Z_g^{(n)})] - \mu_g, \\ \nabla_{\sigma_g} \tilde{L}(\Theta, \theta; Z_g) = \frac{1}{N} \sum_{n=1}^{N} [\nabla_{\sigma_g} \log p_\theta(L_g|Z_g^{(n)}) \\ \qquad + \nabla_{\sigma_g} \log p(F_g|Z_g^{(n)})] + \frac{K(1-\sigma_g)}{2\sigma_g}. \end{cases}$$

(15)

Eventually, optimization of the weight and bias of VAE modules is performed through backpropagation in a conventional manner. The gradient ascent iteration, $\mu_g^{(t+1)} = \mu_g^t + \eta_\mu \nabla_{\mu_g} \tilde{L}(\Theta, \theta; Z_g)$ and $\sigma_g^{(t+1)} = \sigma_g^t + \eta_\sigma \nabla_{\sigma_g} \tilde{L}(\Theta, \theta; Z_g)$, is applied, where η_μ and η_σ is the learning rate.

MLE algorithm for graphical model parameter optimization

To optimize the parameters Θ of the graphical model part in Dynamic-PheSeq with the trained VAE, MLE is applied.

Given the joint probability distribution in equation (13) with the standard mean-field variational inference method, we assume that the latent variables, T, F, follow a variational distribution under pdf $q(T, F)$. The decomposition rule yields to $q(T,F) = \prod_{g=1}^{G} q_g(T_g, F_g)$. In addition, the computation trick in the mean-field variational inference method also derives the optimization of $q_g(T_g, F_g)$ as below:

$$
\begin{aligned}
&\log q_g^*(T_g, F_g) = E_{\prod_{i\neq g}^{G} q_i(T_i,F_i)}[\log p(P,T,F,Z,L|\Theta)] \\
&\quad = \textstyle\sum_i^G E_{\prod_{i\neq g}^{G} q_i(T_i,F_i)}[T_i \log \alpha_i + T_i(\alpha_i - 1)\log P_i \\
&\qquad + (T_i + \alpha_i - 1)\log F_i + \log \frac{\Gamma(a_i+b_i)}{\Gamma(a_i)\Gamma(b_i)} \\
&\qquad + (b_i - T_i)\log(1 - F_i) + \log p_\theta(L_g|Z_g) + \log p(Z_g)] \\
&\quad = T_g \log \alpha_g + T_g(\alpha_g - 1)\log P_g + (T_g + a_g - 1)\log F_g \\
&\qquad + (b_g - T_g)\log(1 - F_g) + C.
\end{aligned}
$$

Henceforth, we have

$$
q_g^*(T_g, F_g) = \alpha_g^{T_g} P_g^{T_g(\alpha_g - 1)} F_g^{(T_g+\alpha_g-1)} (1 - F_g)^{(b_g - T_g)} C',
$$

while $C' = \exp(C)$.

Given the joint probability distribution in equation (13), the logarithm of the joint probability function is

$$
\begin{aligned}
&\log p(P,T,F,Z,L|\Theta) \\
&= \log \textstyle\prod_{g=1}^{G} p(P_g, T_g, F_g, Z_g, L_g|\Theta) \\
&= \textstyle\sum_{g=1}^{G} [T_g \log \alpha_g + T_g(\alpha_g - 1)\log P_g \\
&+ (T_g + a_g - 1)\log F_g \quad + (b_g - T_g)\log(1 - F_g) \\
&+ \log \frac{\Gamma(a_g+b_g)}{\Gamma(a_g)\Gamma(b_g)} + \log p_\theta(L_g|Z_g) \\
&\quad + \log p(Z_g)].
\end{aligned}
$$

For the MLE algorithm, the goal is to maximize the likelihood function. Taking the variational sampling of the latent variable into consideration, a loss function is defined as the expectation of the logarithm of the joint pdf.

$$
L(\phi) = E_{q(T,F)}\left[\log p(P,T,F,Z,L|\Theta)\right] \qquad (16)
$$

A Monte-Carlo estimation of (16) leads to

$$
\widetilde{L}(\phi) = \log\log p(P, T^*, F^*, Z, L|\Theta), \text{ where } T^*, F^* \sim q^*(T, F).
$$

As $\nabla_\phi L(\phi) \approx \nabla_\phi \widetilde{L}(\phi)$, we have the noisy estimate of the gradient with respect to the neural network parameters, ϕ.

$$
\nabla_\phi \widetilde{L}(\phi) \;=\; \sum_{g=1}^{G} (\nabla_{\alpha_g} \tfrac{\partial \alpha_g}{\partial \phi} + \nabla_{a_g} \tfrac{\partial a_g}{\partial \phi} + \nabla_{b_g} \tfrac{\partial b_g}{\partial \phi}),
$$

where

$$
\begin{cases}
\nabla_{\alpha_g} = T_g^*(\log P_g + \frac{1}{\alpha_g}) \\
\nabla_{a_g} = \frac{\Gamma(a_g+b_g)\Psi(a_g+b_g) - \Gamma(a_g+b_g)\Psi(a_g)}{\Gamma(a_g+b_g)} + \log F_g^* \\
\nabla_{b_g} = \frac{\Gamma(a_g+b_g)\Psi(a_g+b_g) - \Gamma(a_g+b_g)\Psi(b_g)}{\Gamma(a_g+b_g)} + \log(1 - F_g^*).
\end{cases}
\qquad (17)
$$

Here, $\Psi(x) = \frac{\Gamma(x)'}{\Gamma(x)}$ is the digamma function. Eventually, a gradient ascent iteration, $\phi^{(t+1)} = \phi^{(t)} + \eta \nabla_\phi \widetilde{L}(\phi)$, is applied, where η is the learning rate.

Pseudocodes of the solution to Dynamic-PheSeq

The iteration of the model parameters is mainly based on formula (15) and (17). The pseudocodes for a solution to the Dynamic-PheSeq model is shown in Algorithm 2.

Algorithm 2 MAP-MLE for Dynamic-PheSeq

Require: Annotated text of G genes: L_G, GWAS p-value of G genes: P_g, Time step: Time

Ensure: optimal ϕ^*, optimal α_g^*, optimal μ_g^*, optimal σ_G^*

Initiate $\alpha^{(0)}$, $\phi^{(0)}$, $\theta^{(0)}$;
Run neural network $f_\phi(Z_g)$ and VAE module $\mathcal{V}_\theta(L_g)$;
for $t = 0$ to $Time$ **do**
  for each gene g **do**
    $Z_g = \mathcal{V}_{\theta^t}(L_g)$;
    $a_g, b_g = f_{\phi^t}(Z_g)$;
    $F_g \sim Beta\,(a_g, b_g)$
    $T_g \sim Bernoulli\,(F_g)$
    $P_g \sim T_g Beta\left(\alpha^{(t)}, 1\right) + (1 - T_g)\,U\,(0,1)$
    $F_g \leftarrow \frac{T_g + a_g - 1}{a_g + b_g - 1}$
    ∇_{μ_g} calculation
    ∇_{σ_g} calculation
    $\mu_g^{(t+1)} = \mu_g^{(t)} - \eta \nabla_{\mu_g}$
    $\sigma_g^{(t+1)} = \sigma_g^{(t)} - \eta \nabla_{\sigma_g}$
    ∇_α calculation
    $\alpha^{(t+1)} = \alpha^{(t)} - \eta \nabla_\alpha$
    $\nabla_{a_g}, \nabla_{b_g}, \frac{\partial a_g}{\partial \phi^{(t)}}, \frac{\partial b_g}{\partial \phi^{(t)}}$ calculation
    $\phi^{(t+1)} = \phi^{(t)} - \eta \nabla_\phi$
  end for
end for

Implementation of PheSeq for Case Studies and General Cases

PheSeq is able to be extent to a good variety of genotype-phenotype association in general cases, out of users' own interest.

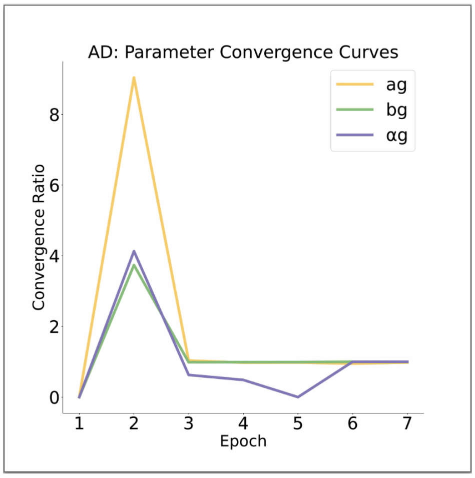

(a)

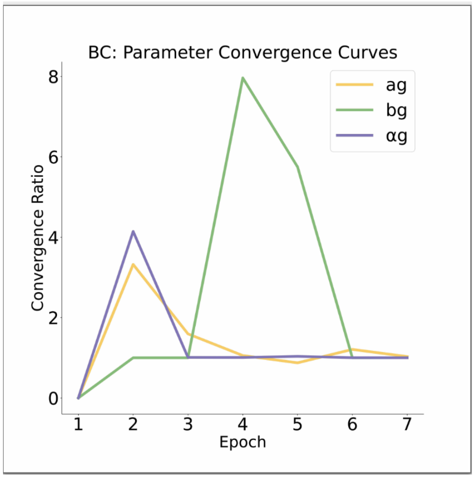

(b)

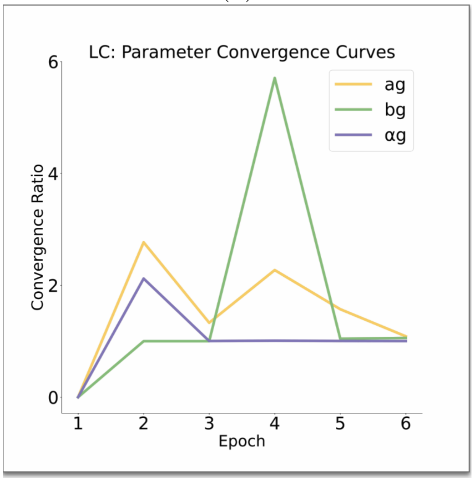

(c)

Figure S3: **Convergence curves of PheSeq parameters in model training. (a)** curve for AD; **(b)** curve for BC; **(c)** curve for LC. To observe the convergence of the PheSeq model, all latent variables, including a_g, b_g, and α_g, are shown in yellow, green, and blue, respectively. The convergence of a parameter θ is denoted as a convergence_ratio $\theta^{(t)}/\theta^{(t-1)}$. The convergence is achieved if the convergence_ratio falls in the interval 1±0.05.

First, users are able to apply standard representation learning methods, such as text embedding for literature data and network data for knowledge graphs, to collect semantic representation for gene embeddings meeting users' own requirement. Second, the significance p-values from conventional sequence analysis are collected accordingly.

The PheSeq model is implemented under a PyTorch framework, where NumPy, math, and sympy packages are used to construct the probability distributions and implement the MLE algorithm. It is suggested that the model is trained with a learning rate of 5e-4, an embedding size of 128, a train time of 100, a p-value threshold of 5e-3, and a batch size of 128. This parameter setting is also suggested to be the default for the user's own experiments. The iterations meet the stopping criterion when all the model parameters achieve convergence. An example of a convergence plot in case studies is provided in **Additional file 3: Fig.** S3, where the model achieves convergence w.r.t all latent variables, including a_g, b_g, and α_g.

To facilitate PheSeq implementation for more disease cases, pre-processed text and embedding data for 32 types of Pan-Cancers in the TCGA database are offered on the project page, `http://lit-evi.hzau.edu.cn/PheSeq/more-diseases`. The data include rich-annotated phenotype descriptions for gene-cancer associations and pre-computed phenotypic embedding for each association pair.
