# Supplementary material for: PheSeq, a Bayesian deep learning model to enhance and interpret the gene-disease association studies
Source: Genome Med. 2024 Apr 16;16:56. doi: 10.1186/s13073-024-01330-7 (PMC11020195; doi:10.1186/s13073-024-01330-7)
Supplement: Supplementary file 3 — Additional file 3. Phenotype Description by PheSeq and A Visualized Phenotype Description Network for AD, BC, and LC. This file includes the embedding visualization of phenotype descriptions utilized in PheSeq. Additionally, it also introduces the web service for visualizing the phenotype description network mentioned in the paper. [file 13073_2024_1330_MOESM3_ESM.pdf]

Additional file 3: Phenotype Description by PheSeq and A Visualized Phenotype Description Network for AD, BC, and LC.

Semantic Partitioning of the Concept-embeddings

To generate the phenotypic embeddings for all gene-disease associations in the three case study diseases, we developed the phenotypic embedding generation pipeline to leverage phenotype concept, sentence description, and network information in terms of phenotype description.

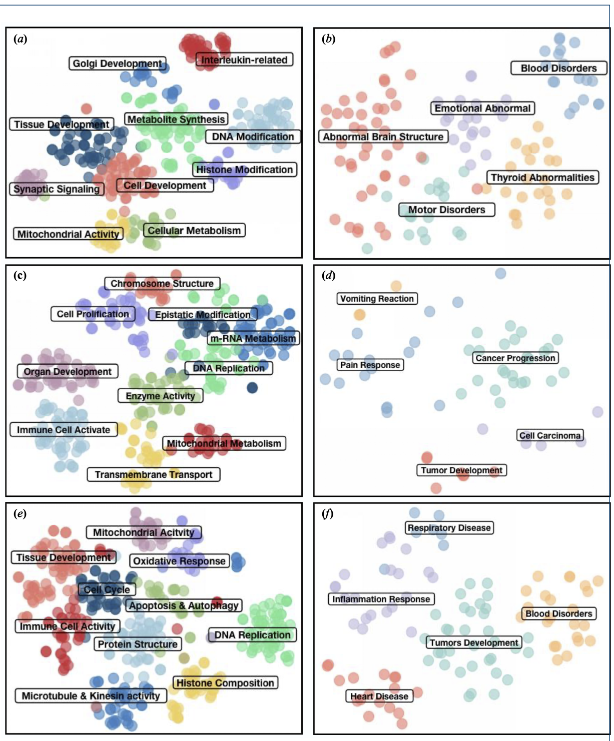

Figure S1: **Visualization of semantic partitions of GO and HPO concept embeddings. (a)** Ten AD-related GO terms, including tissue development, Golgi development, DNA modification, and so on; **(b)** Five AD-related HPO terms, including motor disorders, thyroid abnormalities, structural brain abnormalities, and so on; **(c)** Ten BC-related GO terms, including epistatic modifications, mRNA-related, immune cell activity, and so on; **(d)** Five BC-related HPO terms, including cancer progression, vomiting reaction, and so on; **(e)** Ten LC-related GO terms, including cell cycle, microtubule and kinesin activity, protein structure, and so on; **(f)** Five LC-related GO terms, including tumor development, blood disorders, inflammation response.

As GO and HPO concepts are fundamental parts of the phenotype description, the semantic partitioning of these concept embeddings is observed first to assess the quality of the phenotypic embedding. Here, categories of GO terms and HPO terms are projected to a semantic space by the dimension reduction algorithm of (t-distributed stochastic neighbor embedding, t-SNE).

We randomly selected ten AD-related GO term clusters, i.e., Golgi development, interleukin-related, tissue development, metabolite synthesis, DNA modification, histone modification, synaptic signaling, cell development, mitochondrial activity, and cellular metabolism. After deriving the concept-embedding of the above terms via the phenotypic embedding generation pipeline, we again apply t-SNE to observe the semantic partitioning of these embeddings. As shown in **Additional file 3: Fig.** S1(a), the visualizations exhibit clear semantic partitioning, with different types of phenotype concepts tending to cluster together.

Similarly, five other AD-related HPO term clusters with their semantic partitions are observed in **Additional file 3: Fig.** S1(b). Furthermore, 10 GO term clusters and 5 HPO term clusters are observed for BC and LC in **Additional file 3: Fig.** S1(c-f).

It is noted that the selection of concept terms clusters appears to be random, with varying degrees of relevance to their corresponding diseases. However, regardless of this heterogeneity, there is a consistent adherence to the semantic partitioning of concept embeddings across all cases. The observations indicate that the embeddings maintain well-clustered semantic information, demonstrating the good quality of the semantic perception ability of the phenotypic embedding.

Statistics of Phenotype Description in Three Case Studies

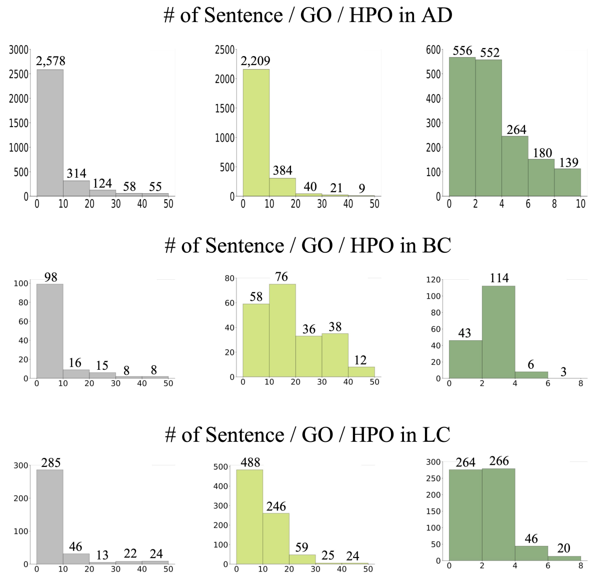

Figure S2: **Histogram of concept and sentence level phenotype descriptions for AD, LC, and BC.**

To Link PheSeq genes with Phenotype description, all GO and HPO evidence supports are derived to form a visualized phenotype description network. There are 14,084, 2,250, and 10,440 pieces of sentence evidence for AD, LC, and BC separately, and the count for each

Table S1: **Statistics information of phenotype descriptions analysis**

	# of phenotype descriptions		
	Sentences	GO terms	HPO terms
AD	14,084	1,849	1,351
BC	2,250	1,075	27
LC	10,440	1,696	170

gene follows a long-tail distribution, as suggested in **Additional file 3: Fig.** S1 and **Additional file 3: Table** S2. For most genes, the association evidence is supported by 10 literature sentences, 10 GO terms, and 2∼4 HPO terms.

The Visualized Phenotype Description Network for Gene Disease Association

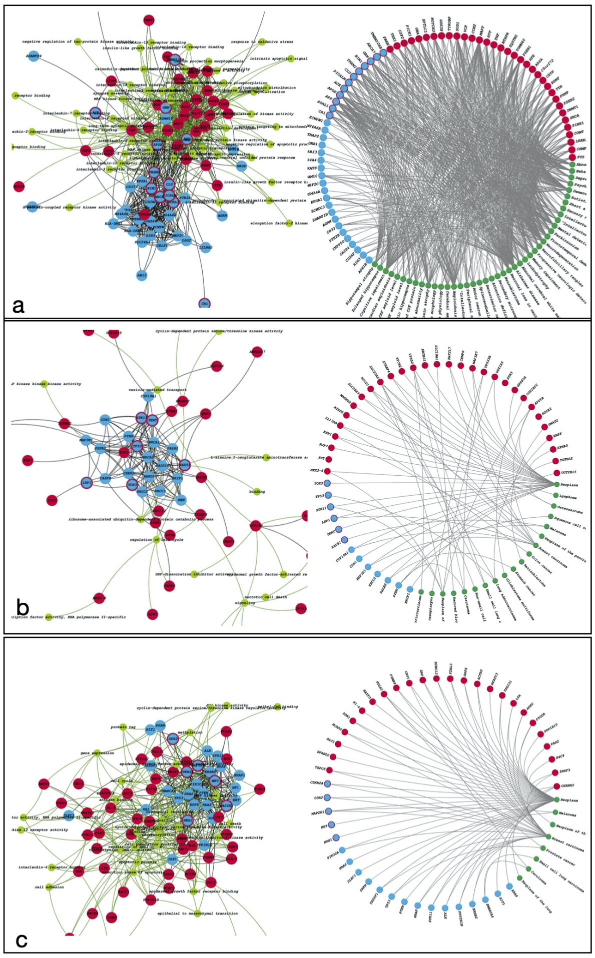

Figure S3: **Web page of the visualized phenotype description network** (http://lit-evi.hzau.edu.cn/PheSeq/AD-Gene-HPO/). **(a) AD page. (b) BC page. (c) LC page.**

To facilitate the illustration of the associations between genes and a given disease, a visualized phenotype description network for all three diseases is constructed by using the co-occurrence of genes and phenotype descriptions. The network is released via a user-friendly visualization web service, `http://lit-evi.hzau.edu.cn/PheSeq`, as shown in **Additional file 3: Fig.** S3.

In adherence to a visualization strategy, genes identified through sequence analysis have been designated blue, while those derived via PheSeq are depicted in red. In instances where these two sources of gene identification overlap, the resulting gene is represented by a red circle with a blue edge. In addition, light green and dark green represent GO terms and HPO terms, respectively. Connecting gene and phenotype descriptions in the network is beneficial for understanding the complex regulatory mechanisms among genes, phenotypes, and gene-disease associations.

The Top 50 AD-associated genes from PheSeq prioritization and 44 genes from IGAP GWAS are all visualized on this page. The gene-AD associations are interpreted through 68 GO terms and 42 HPO terms, augmented by abundant literature evidence. Similarly, 50 PheSeq genes and 26 Agilent G4502A_07_3 sequence analysis genes are associated with 50 GO and 8 HPO for BC, and 50 PheSeq genes and 17 HumanMethylation450 sequence analysis genes are associated with 50 GO and 11 HPO for LC.

The establishment of connections between gene and phenotype descriptions within the network is a valuable tool for improving our comprehension of the intricate regulatory mechanisms governing genes, as well as facilitating investigations into gene-disease associations.

In the network, prioritized genes from PheSeq, sequence analysis, and both sources are represented, and the phenotype descriptions are traceable through web browsing. Benefiting from these abundant phenotype descriptions by GO and HPO terms, the associations between prioritized genes and disease are interpreted in in-depth phenotype descriptions, and a variety of knowledge inference patterns are suggested. First, we provide a GO enrichment analysis. The GO-linked gene set is enriched in the corresponding molecular mechanism. Second, we allow observation of genes both from PheSeq and sequence analysis. Adding PheSeq-prioritized genes (marked as red circles) allows further investigation of the gene impact on pathology at a molecular level. Third We provide a hybrid investigation of gene-phenotype associations. Here, co-occurring genes in phenotype descriptions are prone to unveil gene-gene interactions. Integrative analysis of multiple GO terms leads to the discovery of complex gene pathological pathways. Linking the gene-GO and gene-HPO associations contributes to interpreting a multi-level pathology mechanism. Finally, an evidence-supported gene-GO network contributes to integrating the findings, pinpointing vital disease-associated genes. We also integrate PPI info into the network but exclude the external GO/HPO annotation taking into account the noise. The considerations are provided in the following.

Auxiliary PPI info in the visualized phenotype description network

We present several instances of hub nodes and common nodes in the PPI network in Additional file 3: Fig. S4 and Additional file 3: Fig. S5, respectively, and compare their different scenarios in association connections. Without exception, the genes illustrated in these examples are identified as significant genes by PheSeq.

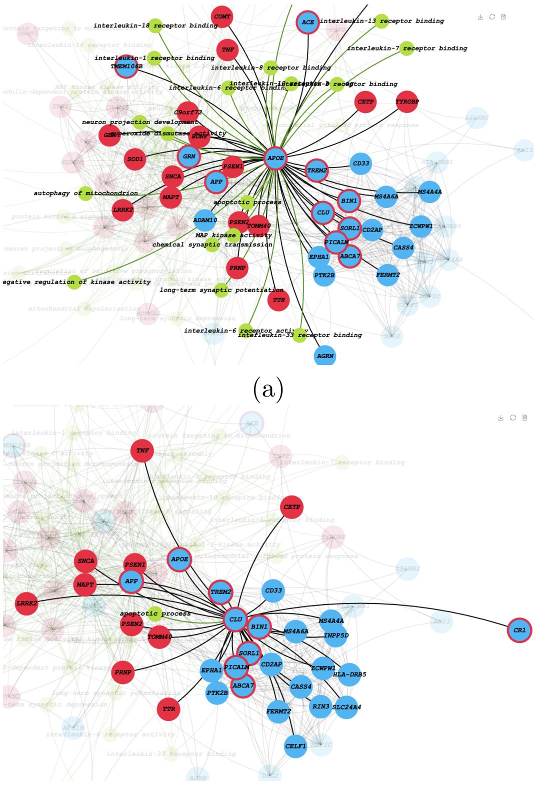

(a)

(b)

Figure S4: **Hub gene nodes in PPI network with association connections. (a/b) Hub genes with/without abundant phenotype links.**

In Additional file 3: Fig. S4, the APOE node exhibits an abundance of external PPI links (depicted by black edges) and a rich array of phenotype links (depicted by green edges). Conversely, the node CLU predominantly serves as a hub through PPI connections. Consistent with the patterns observed in the ablation experiments, a node is likely identified as a significant gene (depicted by red circles or blue circles with red borders) by PheSeq if it is prominently connected to other significant genes or phenotype descriptions.

Additional file 3: Fig. S5 presents three distinct common gene nodes, each exhibiting shared linkage attributes. Firstly, they do not serve as hub nodes with numerous connections. Secondly, they have limited associations with external phenotype links. Specifically, the CEPT gene in (a) and TYROBP gene in (b) are each associated with only one phenotype, while the TOMM40 gene in (c) is associated with merely three phenotypes. Lastly, they are all connected to a good quantity of key genes in the PPI network, enhancing the significant attributes in embedding values. Eventually, PheSeq identifies these three genes as significant genes.

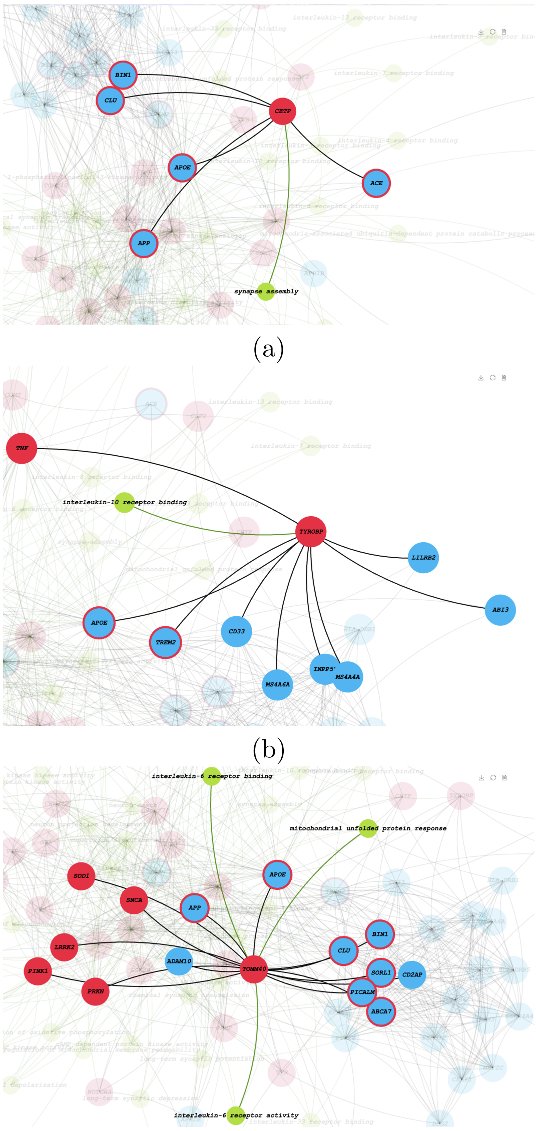

(a)

(b)

(c)

Figure S5: **Common gene nodes in PPI network with association connections. (a) CEPT. (b) TYROBP. (c) TOMM40.**

Consideration of auxiliary GO/HPO annotations in the visualized phenotype description network

While the external GO and HPO annotations enable an extensive set of external gene information, they are not yet integrated into the visualized phenotype description network.

On one hand, a portion of additional GO/HPO annotations assists in providing explanations. For instance, in GO annotation, BIN1 is associated with GO:0045664 'regulation of neuron differentiation', PRKN is annotated to GO:0070050 'neuron cellular homeostasis', and SNCA is annotated to GO:0051402

'neuron apoptotic process'. Similarly, in HPO annotation, TMEM106B is annotated to HP:0002354 'Memory impairment', C9orf72 is annotated to HP:0030191 'Abnormal peripheral nervous system synaptic transmission', and MAPT is annotated to HP:0002450 'Abnormal motor neuron morphology'.

These GO annotations, overlooked by PheSeq, may assist in further complementing the network interpretation of gene-pathology relationships. However, the majority of the remaining GO/HPO annotations lack specificity to a particular disease context and may introduce noise to the network interpretation. (In PheSeq, the gene-GO/HPO annotations are collected in a disease-specific context.)

For example, in GO annotation, PSEN1 is annotated to GO:0043589 'skin morphogenesis', APOE is annotated to GO:0010629 'negative regulation of gene expression', and TREM2 is annotated to GO:0035176 'social behavior'. In HPO annotation, MAPT is annotated to HP:0100738 'Abnormal eating behavior', APP is annotated to HP:0003011 'Abnormality of the musculature', and C9orf72 is annotated to HP:0001315 'Reduced tendon reflexes'. These GO/HPO annotations seem unrelated to the AD disease context and their inclusion in the network may introduce noise to the interpretation of gene pathology.
